# Genomic Survey of Pathogenicity Determinants and VNTR Markers in the Cassava Bacterial Pathogen *Xanthomonas axonopodis* pv. Manihotis Strain CIO151

**DOI:** 10.1371/journal.pone.0079704

**Published:** 2013-11-22

**Authors:** Mario L. Arrieta-Ortiz, Luis M. Rodríguez-R, Álvaro L. Pérez-Quintero, Lucie Poulin, Ana C. Díaz, Nathalia Arias Rojas, Cesar Trujillo, Mariana Restrepo Benavides, Rebecca Bart, Jens Boch, Tristan Boureau, Armelle Darrasse, Perrine David, Thomas Dugé de Bernonville, Paula Fontanilla, Lionel Gagnevin, Fabien Guérin, Marie-Agnès Jacques, Emmanuelle Lauber, Pierre Lefeuvre, Cesar Medina, Edgar Medina, Nathaly Montenegro, Alejandra Muñoz Bodnar, Laurent D. Noël, Juan F. Ortiz Quiñones, Daniela Osorio, Carolina Pardo, Prabhu B. Patil, Stéphane Poussier, Olivier Pruvost, Isabelle Robène-Soustrade, Robert P. Ryan, Javier Tabima, Oscar G. Urrego Morales, Christian Vernière, Sébastien Carrere, Valérie Verdier, Boris Szurek, Silvia Restrepo, Camilo López, Ralf Koebnik, Adriana Bernal

**Affiliations:** 1 Laboratorio de Micología y Fitopatología Uniandes (LAMFU), Universidad de Los Andes, Bogotá, Colombia; 2 Unité Mixte de Recherche Résistance des Plantes aux Bioaggresseurs, Institut de Recherche pour le Développement, Montpellier, France; 3 Manihot-Biotec, Departamento de Biología, Universidad Nacional de Colombia, Bogotá, Colombia; 4 Department of Plant and Microbial Biology, University of California, Berkeley, California, United States of America; 5 Department of Genetics, Martin Luther University, Halle-Wittenberg, Germany; 6 Institut National de la Recherche Agronomique, UMR45 Institut de Recherche en Horticulture et Semences, Beaucouzé, France; 7 Université d'Angers, UMR1345 Institut de Recherche en Horticulture et Semences, SFR4207 Quasav, PRES L'UNAM, Beaucouzé, France; 8 Agrocampus Ouest, UMR1345 Institut de Recherche en Horticulture et Semences, Beaucouzé, France; 9 Laboratoire des Interactions Plantes Micro-organismes (LIPM), UMR 441, Castanet-Tolosan-Microorganismes, Institut National de la Recherche Agronomique. Toulouse, France; 10 Laboratoire des Interactions Plantes Micro-organismes (LIPM), UMR 2594, Centre National de la Recherche Scientifique, Castanet-Tolosan, France; 11 Unite Mixte de Recherche Peuplement Végétaux et Bioagresseurs en Milieu Tropical, Centre de coopération internationale en recherche agronomique pour le développement, La Réunion, France; 12 Institute of Microbial Technology, Council of Scientific and Industrial Research, Chandigarh, India; 13 College of Life Sciences, University of Dundee, Dundee, Scotland; 14 Department of Bioagricultural Sciences and Pest Management, Colorado State University, Fort Collins, Colorado, United States of America; University of the West of England, United Kingdom

## Abstract

*Xanthomonas axonopodis* pv. *manihotis* (*Xam*) is the causal agent of bacterial blight of cassava, which is among the main components of human diet in Africa and South America. Current information about the molecular pathogenicity factors involved in the infection process of this organism is limited. Previous studies in other bacteria in this genus suggest that advanced draft genome sequences are valuable resources for molecular studies on their interaction with plants and could provide valuable tools for diagnostics and detection. Here we have generated the first manually annotated high-quality draft genome sequence of *Xam* strain CIO151. Its genomic structure is similar to that of other xanthomonads, especially *Xanthomonas euvesicatoria* and *Xanthomonas citri* pv. *citri* species. Several putative pathogenicity factors were identified, including type III effectors, cell wall-degrading enzymes and clusters encoding protein secretion systems. Specific characteristics in this genome include changes in the xanthomonadin cluster that could explain the lack of typical yellow color in all strains of this pathovar and the presence of 50 regions in the genome with atypical nucleotide composition. The genome sequence was used to predict and evaluate 22 variable number of tandem repeat (VNTR) loci that were subsequently demonstrated as polymorphic in representative *Xam* strains. Our results demonstrate that *Xanthomonas axonopodis* pv. *manihotis* strain CIO151 possesses ten clusters of pathogenicity factors conserved within the genus *Xanthomonas*. We report 126 genes that are potentially unique to *Xam*, as well as potential horizontal transfer events in the history of the genome. The relation of these regions with virulence and pathogenicity could explain several aspects of the biology of this pathogen, including its ability to colonize both vascular and non-vascular tissues of cassava plants. A set of 16 robust, polymorphic VNTR loci will be useful to develop a multi-locus VNTR analysis scheme for epidemiological surveillance of this disease.

## Introduction

The genus *Xanthomonas* comprises plant pathogens that infect a wide range of plants, including citrus, pepper, tomato, rice and others [1], [2]. In particular, *Xanthomonas axonopodis* pv. *manihotis* (*Xam*) is the causative agent of cassava bacterial blight (CBB; [3]), the main bacterial disease affecting cassava plants (*Manihot esculenta*). This disease is widespread in all places where cassava is grown, including Africa, Asia and South America [4], [5], where crop losses due to CBB have been reported to be between 12 and 100% [3]. Accordingly, the journal *Molecular Plant Pathology* recently listed *Xam* among the top 10 plant pathogenic bacteria based on its scientific and economic importance [6].

*Xam* is mainly a vascular pathogen and survives epiphytically until favorable conditions for infection are reached. Open wounds and stomata are major routes of pathogen infection in otherwise healthy plants [4], and the disease is transmitted between crop cycles through the use of infected cuttings [7]. Once inside the plant, bacteria colonize the mesophyll, generating angular leaf spots as one of the early symptoms. In subsequent stages in susceptible plants, pathogen population increases and reaches vascular tissues, blocking the flow of nutrients and generating a wilting process that, in severe cases, ends with the death of the plant [3]. Alternatively, when infection starts by the use of infected propagative material, it spreads immediately in the vascular tissues, leading to a rapid wilt of the plant (reviewed by [7]). Control strategies to prevent CBB spread include the use of resistant cassava varieties and pathogen-free plant cuttings [3]. Nonetheless, the molecular basis of resistance is not completely understood and it is permanently challenged by the diversity of *Xam* strains [8], [9]. Also, knowledge on the early determinants of disease development is limited. A better understanding of the pathogenicity mechanisms of *Xam* at the molecular level is urgently needed to efficiently control this disease.

Among the most important pathogenicity factors are the diverse protein secretion systems and their substrates [10]. Of special interest are type III-secreted effector proteins (T3E), which play an important role in the plant-pathogen interaction and in shaping the host range [11], [12], [13]. Moreover, conserved T3E in *Xanthomonas* have been proposed as an ancestral characteristic for pathogenicity and virulence inside the genus [14]. About twenty potential T3Es per genome have been identified in different *Xanthomonas*, with more than sixty different potential T3E found among all the bacteria of this genus [13]. More recently, 22 effector gene families were reported to be present in several genomic sequences of *Xam* [15]. Despite this wealth of information, in *Xam*, only one T3E, belonging to the TAL effector family has been reported as a virulence factor [16]. Several other elements such as exopolysaccharides (EPS) and cell wall degrading enzymes (CWDE), with attributed pathogenicity and virulence roles in other xanthomonads, might be important for *Xam* [17], [18]. In fact, for this particular pathogen, the synthesis of EPS has been reported as a virulence factor [19]. However, in order to comprehensively characterise the pathogenicity repertoire of plant pathogens such as *Xam* it is necessary to move beyond single gene approaches and to apply genomics tools. Further, genomics approaches may reveal origins of pathogenicity and virulence factors and thus contribute to our understanding of how microbial pathogenesis evolves.

Virulence factors are microbial adaptations and can arise from *de novo* mutations or through gene flow among populations or species. In recent years, the importance of horizontal gene transfer (HGT) events that lead to the acquisition of foreign DNA sequences has been well documented in bacteria [20], [21], [22]. Efforts to define the impact of these events in the genomic structure and variations between closely related species have been made [23]. For example, a foreign origin of the type III secretion system (T3SS) has been proposed in the genus *Xanthomonas* [24], and contribution of HGT to the genome composition has been measured in *X. citri* pv. *citri* str. 306 (*Xac*) (syn. *X. citri* subsp. citri) and *X. campestris* pv. *campestris* str. ATCC 33913 (*XccATCC*) species [23], [25]. However, the determination of HGT events in *Xam*, as well as their contribution to pathogenicity is still missing.

Efficient control of CBB will also critically depend on a profound knowledge of the population structure of *Xam* in different regions of the world. Traditionally, bacterial isolates have been typed by various fingerprinting techniques [26], [27], [28], [29], [30], [31]. Since then, multiple loci variable number of tandem repeat (VNTR) analysis (MLVA) has become increasingly popular for molecular typing of bacteria [32], [33]. MLVA is a method for molecular typing of bacterial strains that explores the natural variation in the number of tandemly repeated DNA sequences. MLVA has several advantages over other bacterial genotyping methods, such as ease of performance and portability, high reproducibility and discriminatory power, rapidity and low costs [34]. Powerful MLVA schemes are available for most important bacterial pathogens infecting humans, including *Bacillus anthracis*, *Escherichia coli*, *Mycobacterium tuberculosis*, *Pseudomonas aeruginosa* and *Yersinia pestis* [32], [35]. With the advent of genomics, development of powerful MLVA schemes became a straightforward procedure [34]. Consequently, the first VNTR study of a bacterial plant pathogen, *Xylella fastidiosa*, was published in 2001 [36], and VNTR schemes are now available for *Candidatus Liberibacter asiaticus*, *Pseudomonas syringae* and several pathovars of *Xanthomonas* [37], [38], [39], [40], [41].

Here, we report the first manually annotated high-quality draft genome sequence of *Xam* strain CIO151, obtained by 454 sequencing technology, and analysis of the impact of presumed HGT events on the gene content of *Xam*. We defined a set of potential pathogenicity determinants through a comparative genomic approach and report sets of genes encoding for T3E proteins, cell wall-degrading enzymes, secretion systems, among others; thus obtaining a deeper insight into the gene repertoire of *Xam* CIO151. This strain was selected for sequencing for several reasons: (i) QTL mapping of resistance markers effective against CIO151 has been carried out in a cassava mapping population [42]. (ii) Aggressiveness of this strain against several cassava cultivars has been measured [9]. (iii) Effects of CIO151 inoculation on gene expression of cassava have been characterized [43], [44]. This sequence provides an efficient approach to understand the pathogenicity of this organism and to develop future studies oriented at increasing the knowledge on the biology of *Xam* and the cassava-*Xam* interaction. Finally, we took advantage of this new genomic resource to develop an inexpensive and user-friendly molecular typing tool based on VNTR loci.

## Results

### General features

The draft genome sequence of *Xanthomonas axonopodis* pv. *manihotis* (*Xam*) strain CIO151 was produced with 454 sequencing technology. This strain had recently been sequenced with Illumina technology [15] for the identification of type III effectors and the determination of phylogenetic relationships of *Xam* strains around the world. Though adequate for these aims, the previously reported draft genome assembly was not sufficient for a comprehensive genome mining and comparative genomics. A total of 305,637 clipped base-called reads with a median read length of 359 bases and coverage of 21.9× were obtained. The genome sequence is composed of 36 scaffolds with a total length of 5.15 Mb, a gene coding capacity of 82.8% and a N50 scaffold size of 429.5 kb. The sequence has a high G+C content (65.1%), as commonly reported for the genus *Xanthomonas* [45]. A total of 4340 putative coding sequences (CDS), two rRNA operons, and 55 tRNA genes for all amino acids were identified (**Table 1**). Upon automatic annotation, an international consortium of scientists with expertise on different aspects of the biology of *Xanthomonas* manually annotated all predicted genes. This manual annotation led to modifications of structural annotation (860 CDS) and functional annotation (3818 CDS). In addition, 369 predicted CDS were removed.

**Table 1:** General features of the genome of *Xam* CIO151.

Features of the genome assembly of *Xam* CIO151	
Size (bp)	5,150,225
Number of scaffolds	36
G+C (%)	65.1
Insertion sequences and transposons	250
Regions with atypical composition	62
Predicted CDS	4340

RNA	
tRNAs	55
rRNA operons	2

The gaps on the genome sequence did not preclude the identification and analysis of conserved gene clusters and potential pathogenicity factors. *Xam* CIO151 shares a number of genomic features with other members of the genus. Yet, we could detect differences that could be relevant for its biology, including variations in some T3E, the xanthomonadin cluster and regions putatively associated with HGT events.

**Figure 1:**
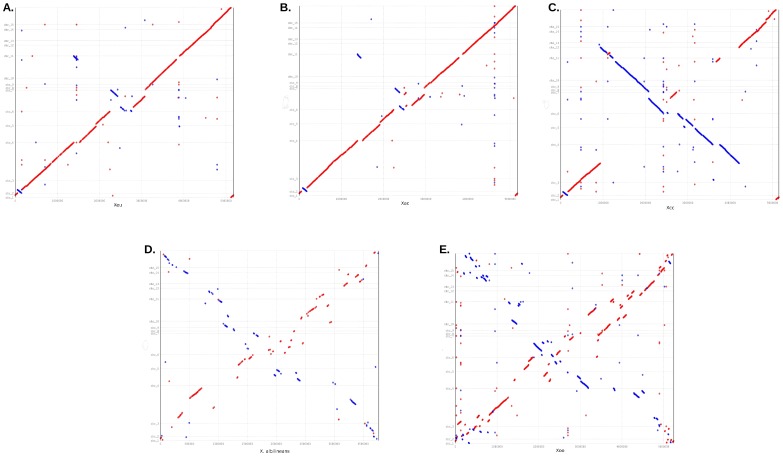
Comparison of the genomic structure of *Xam* CIO151 with that of closely related members from the genus *Xanthomonas*. Scaffolds of *Xam* CIO151 were ordered based on the alignment with the complete genome sequence of *X. euvesicatoria*, ***Xeu***, and then genome comparisons were performed using MUMmer (**A**). Alignment of ordered scaffolds of *Xam* CIO151 with the complete genome sequences of *X. axonopodis* pv. citri str. 306, ***Xac*** (**B**); *X. campestris* pv. campestris str. 8004, ***Xcc*** (**C**); *X. albilineans*, ***Xal*** (**D**); and *Xanthomonas oryzae* pv. *oryzae* PXO99^A^, ***Xoo*** (**E**) chromosomes. Scaffolds classified as parts of the chromosome of *Xam* CIO151 are shown in the y-axis. Red dots represent conserved segments while blue dots represent inverted regions.

Fifteen out of the 36 assembled scaffolds, accounting for 4.82 Mb, could be mapped to the chromosome of *Xanthomonas euvesicatoria* and were classified as part of the chromosome (see methods, **Fig. 1**). As usual, the *dnaA* gene was placed at the beginning of the genome sequence and corresponds to the start of the first scaffold. Scaffold mapping resulted in an asymmetric GC skew pattern which is typical for bacterial genomes [46], thus supporting the proposed order of scaffolds. Scaffolds that could not be mapped were classified in two categories: (i) candidate plasmid sequences of small length, which showed similarity to plasmids of other xanthomonads, and (ii) sequences of unknown origin, which did not show high similarity to chromosomal or plasmid sequences of other xanthomonads. In total, 93.64% of the genome sequence was classified as chromosomal, 1.72% was classified as candidate plasmid sequences and 4.64% was classified as unknown. The proportion of candidate plasmid and chromosomal sequences is in agreement with values reported for other *Xanthomonas* strains, such as *Xac* and *Xeu* [25], [47].

### *Xam* CIO151 shares a similar genome structure with *Xac* and *Xeu*

In order to evaluate the genome structure of *Xam* CIO151 and to compare it with the structure of other xanthomonads, global genomic alignments (**Fig. 1**) and an analysis of collinear blocks at the intrascaffold level were performed using MAUVE software [48] (**Fig. S1**). *Xam* CIO151 structure is most similar to that of *Xac* and *Xeu*, with three small inversions (**Fig. 1A–B**). A large rearrangement was observed in the comparison between *Xam* CIO151 and *Xcc8004* (**Fig. 1C**). Numerous rearrangements are evident between *Xam* CIO151 and *X. oryzae* pv. *oryzae* PXO99^A^ (*XooPXO99A*) [49] and between *Xam* CIO151 and *X. albilineans* (*Xal*) [50] (**Fig. 1D–E**). MAUVE alignments showed an increase in rearrangements of collinear regions in scaffolds that mapped close to the replication terminus, which supports our genome assembly. Only two scaffolds showed rearrangements in consecutive collinear regions in alignments of *Xam* CIO151 with *Xeu* and *Xac*, while more evident rearrangements were observed in the alignments with *Xcc* and *Xoo* (**Fig. S1**). Large duplications were not detected in the set of scaffolds classified as chromosomal regions. In addition, several genomic islands, not shared by both species under comparison (white regions), were also identified (**Fig. S1**).

### Unique proteins of *Xam* CIO151

Differences in host range among taxa in this genus might be associated with the presence or absence of specific pathogenicity determinants in each taxon. Based on this hypothesis, we compared the predicted proteome of *Xam* CIO151 with those of other xanthomonads. In total, 126 proteins were identified as unique to *Xam* (excluding proteins encoded by incompletely sequenced genes) (**Table S1**). Among these 126 proteins, the most frequent matches in BLAST searches against Genbank corresponded to translated sequences from members of the *Burkholderia* genus. In general, a high percentage of the *Xam* CIO151-specific proteins (81%) were annotated as hypothetical or conserved hypothetical proteins and only seven potentially secreted proteins and four putative membrane proteins were identified (**Table S1**). We also evaluated the potential presence of the 126 proteins in the genome sequence of the 65 *Xam* strains reported by Bart and collaborators [15] using a tblastn [51]. There were 58 proteins with hits in all 65 *Xam* strains (covering at least 80% of the sequence and with a similarity of at least 30%). This set of unique proteins is an important starting point in the identification of elements potentially involved in the specific cassava-*Xam* interaction.

### Horizontal gene transfer and pathogenicity islands

Identifying genomic regions potentially acquired by horizontal gene transfer (HGT) events is a key step in understanding the features that are unique to the members within a species, as well as those which contribute to divergence in a given taxon [52], [53]. Using the automatic annotation produced by iANT (integrated ANnotation Tool) [54], a set of 250 putative insertion sequences and transposable (IS/Tnp) elements (including full and fragmented sequences) were identified in all scaffolds.

**Figure 2:**
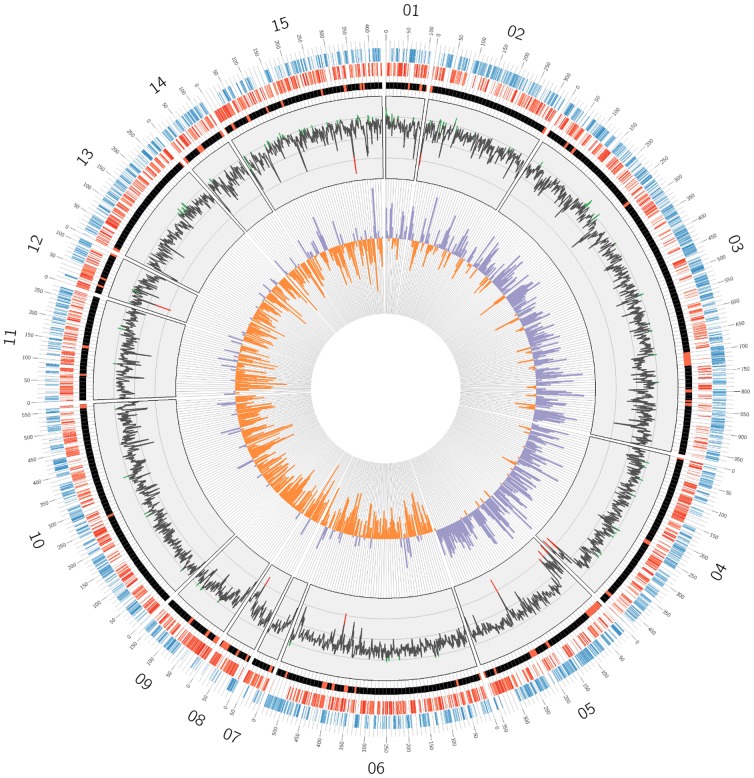
Circular representation of the genome sequence of *Xam* CIO151. From outside to inside: first circle in blue indicates CDS predicted in the positive strands for the scaffolds classified as probable chromosomal regions. Second circle in red indicates the CDS predicted in the negative strand. Red spots in the black third circle indicate the region identified with atypical nucleotide composition. The fourth circle indicates the deviation pattern from the average G+C content. Inner circle shows GC skew values, positive values are shown in purple and negative values are shown in orange. Numbers correspond to scaffold IDs.

Sixty-two candidate regions with atypical nucleotide composition and potentially related to HGT events were identified in the genome sequence of *Xam* using AlienHunter (**Table 1** **;** **Fig. 2** **; Table S2**). The total length of predicted regions was 640 kb (including regions in putative non-chromosomal scaffolds), which corresponds to 12.4% of the total sequence of *Xam* CIO151. The average length of the predicted regions is 10.3 kb and twenty two regions are larger or equal to 10 kb.

Genomic islands resulting from HGT events generally share some characteristics, such as the unusual G+C content, the vicinity of tRNA genes and/or mobile genetic elements, among others [55]. Indeed, 33 out of the 62 regions with atypical nucleotide composition have elements similar to reported mobile sequences (all 33 regions with plasmid sequence and seven with additional prophage derived sequences); 19 are located close to predicted tRNA genes and ten chromosomal elements have both. Additionally, twelve regions cover 50.4% of scaffolds that were not identified as part of the chromosome of *Xam* CIO151. This finding supports the potential alien origin of those scaffolds.

Pathogenicity islands (PAIs) constitute a special class of HGT-acquired regions. In addition to the previously described characteristics of genomic islands, PAIs also contain genes associated with virulence [55]. In order to identify potential PAIs present in the genome of *Xam* CIO151, we scrutinized the genomic islands for genes consistent with functions in virulence.

Four potential PAIs were identified (**Table S3**). The first two contain genes that have been suggested in other xanthomonads to constitute putative PAIs, including a type IV secretion system and genes associated with type IV pili (pilY, pilX, pilW, pilV and fimT) [23], [47]. The third candidate PAI encodes a XopC2 effector and two fragmented versions of the XopP effector. The fourth candidate PAI contains two probable pseudogenes (due to early stop codons) similar to the fhaB adhesin family.

### Mutations in the xanthomonadin cluster could explain the white phenotype of *Xam*

**Figure 3:**
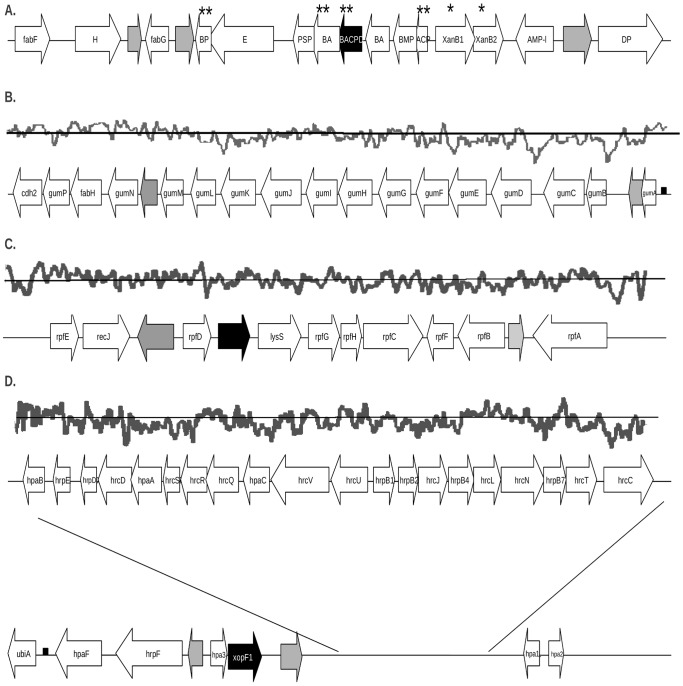
Organization of pathogenicity-related gene clusters in the *Xam* CIO151 genome. Open arrows with labels indicate genes with assigned functions, black arrows indicate genes with early stop codons, open arrows without labels indicate conserved hypothetical proteins and grey arrows indicate non-conserved hypothetical proteins. Graphs above clusters show the G+C content and deviations from the average value. **A**. Xanthomonadin gene cluster; * indicates genes related to *pigB* genomic region and ** indicates genes reported as important for cluster functionality. Abbreviations used are: **H** = halogenase (xanmn_chr15_0075), **BP** = xanthomonadin biosynthesis protein (xanmn_chr15_0079), **E = **xanthomonadin exporter (xanmn_chr15_0373), **PSP** = putative secreted protein (xanmn_chr15_0080), **BACPD** = xanthomonadin biosynthesis acyl carrier protein dehydratase (xanmn_chr15_0082), **BA** = putative xanthomonadin biosynthesis acyltransferase (xanmn_chr15_0081 and xanmn_chr15_0083), **BMP** = putative xanthomonadin biosynthesis membrane protein (xanmn_chr15_0084), **ACP** = acyl carrier protein (xanmn_chr15_0085), **XanB1** = putative reductase/halogenase (xanmn_chr15_0086), **XanB2** = putative pteridine-dependent deoxygenase like protein (xanmn_chr15_0087), **AMP-l** = AMP-ligase (xanmn_chr15_0088), **DP** = dipeptidyl peptidase (xanmn_chr15_0090). **B**. Cluster implicated in xanthan production (*gum*). **C**. Regulation of pathogenicity factors (*rpf*) cluster. **D**. Type III secretion system (**T3SS**) cluster.

Xanthomonadins are yellow pigments, which are diagnostic for xanthomonads (Greek xanthós = yellow) [56]. A role for xanthomonadins in epiphytic survival and protection against photobiological damage has been proposed for *Xcc* strain B24 [57]. However, some xanthomonads lack this pigment, including *Xam*, *X. citri* pv. *mangiferaeindicae* and some strains of *Xcc* [58]. In order to detect changes that could potentially explain the white phenotype of all *Xam* strains, the xanthomonadin cluster (also known as *pig* cluster) was analyzed (**Fig. 3A**). This cluster consists of seven transcriptional units in *Xcc* [58] and encodes fourteen open reading frames (ORFs) in *Xoo* strains [59]. Four of them are required for xanthomonadin biosynthesis, including two ORFs, XanB1 (xanmn_chr15_0086) and XanB2 (xanmn_chr15_0087), located in the *pigB* region [57]. Based on high sequence similarity with homologs in other xanthomonads, most genes seem to be functional in the genome of *Xam* CIO151. Interestingly however, gene xanmn_chr15_0082, which encodes an acyl carrier protein dehydratase, appears to be non-functional in *Xam*, due to a frameshift at position 110 which results in a 50 amino acid protein instead of the 95 residues reported for the *Xeu* predicted protein (**Fig. 3A**). This gene has been reported as an important element for xanthomonadin synthesis in *Xoo* strains [59] and a predicted loss of function in *Xam* could cause the white phenotype of this bacterium. This hypothesis is supported by an analysis of 65 draft genome sequences of *Xam* [15], which shows that the early stop mutation is conserved among strains in the pathovar.

### Several secretion systems, pathogenesis-associated clusters and elements involved in cell-to-cell signaling are conserved in *Xam* CIO151

Cell-to-cell signaling is a key bacterial mechanism for sensing population density levels through diffusible signal molecules [60]. The genome of *Xam* CIO151 carries the *rpf* (for regulation of pathogenicity factors) gene cluster that is found in all xanthomonads and encodes components governing the synthesis and perception of the diffusible signal factor DSF [61], [62]. This cluster is composed of *rpfABCDEFGH* genes (**Fig. 3C** **; Table S4**). The Rpf/DSF system regulates the synthesis of virulence factors and biofilm formation and is required for the full virulence of *Xcc*, *Xac*, *Xoc*, and *Xoo* on their hosts [63], [64], [65], [66], [67]. RpfF is responsible for the synthesis of DSF, whereas RpfC and RpfG comprise a two-component system implicated in DSF perception and signal transduction [61], [68], [69]. RpfC is a complex sensor kinase, while RpfG is a response regulator with a HD-GYP domain that acts in degradation of the second messenger cyclic di-GMP [70]. In addition to genes encoding these products, *Xam* CIO151 encodes for *rpfH*, a membrane protein related to the sensory input domain RpfC with unknown function. RpfH is present in *Xeu* and *Xcc* but absent in *Xac* and *Xoo.*

Bacteria use several secretion systems to secrete a diversity of proteins. *Xam* CIO151 possesses all the protein secretion systems that have been reported so far for other Gram-negative bacteria. The corresponding gene clusters show a conserved synteny to those of *Xac* and *Xeu*. The type II secretion system (T2SS) is important for the secretion of cell wall-degrading enzymes in different plant-pathogens [71], [72], [73]. Two copies of this cluster were detected in the genome of *Xam* CIO151. The *xps* cluster (*xpsEFGHIJKLMND* genes) was detected in a genomic region of 10.4 kb (**Table 2** **& Table S5**). The *xcs* cluster, encoding a second T2SS (*xcsCDEFGHIJKLMN* genes) was identified in another genomic region of 10.8 kb (**Table 2** **& Table S5**). This second T2SS is present in other xanthomonads of the *X. axonopodis* and *X. campestris* species [74].

**Table 2:** Putative pathogenicity elements identified in the genome of *Xam* CIO151.

Functional category	Number of related genes
Type I protein secretion system	5
Type II protein secretion system	
*xps* cluster	11
*xcs* cluster	12
Type III protein secretion system (*hrp* cluster)	28
Type III effector proteins	28
Type IV protein secretion system	22
Type VI protein secretion system	15
Type IV pili	24
Flagellum^1^	32
Regulation of pathogenicity factors (*rpf* cluster)	8
Xanthomonadin	18
Xanthan (*gum* cluster)	16
Cell wall-degrading enzymes	30
Polyketide synthase (PKS)^1^	26
Siderophore biosynthesis^1^	3
Toxins^1^	6
Chemotaxis^1^	47

1 Number indicates CDS identified by key word searches in iANT.

The T3SS encoded by the *hrp* gene cluster is involved in the secretion and translocation of effector proteins and is a key pathogenicity factor in most xanthomonads [75]. In *Xam* CIO151, it is composed of twenty-eight genes, including the *hpaF* effector gene and a putative *xopF* pseudogene, in a genomic region of 27.3 kb (**Fig. 3D** **; Table S6**). Its G+C content is 62.8%, a value slightly lower than the average for this genome and comparable to that of other xanthomonads [74]. *Xam* CIO151 shares all conserved genes of the *hrp* cluster and is predicted to possess two non-conserved hypothetical proteins in this cluster. One of them is located between the *hpaB* and *xopF* genes, and the other one is located between *hpa3* and *hrpF*. Whether or not these genes play a role in pathogenicity awaits experimental confirmation.

The T4SS has been reported to mediate translocation of DNA and effector proteins in bacterial interactions with other bacteria and with their eukaryotic hosts [76], [77]. Two different clusters varying in their gene organization were identified in the sequence of *Xam* CIO151 and they were classified according to Moreira and collaborators [78]. The first one belongs to class I (T4SS[I]). It consists of eleven genes in a genomic region of 12.5 kb and is highly similar to the T4SS from *Legionella pneumophila* [79]. This is reminiscent of the type IV secretion system identified in the plasmid pXCV183 of *Xeu* [47]. Nonetheless, the cluster present in *Xam* differs from that in *L. pneumophila* by the apparent absence of a *virB7* homolog and the presence of an additional incomplete sequence similar to *virB4*. Interestingly, this cluster is located on a probably non-chromosomal 35-kb scaffold with atypical nucleotide composition. It has some mobile elements and is partially similar to plasmid sequences of other xanthomonads and a plasmid from *Methylobacterium radiotolerans*, suggesting that this cluster is located on a plasmid. The second one is composed of eleven genes in a genomic region of 12.8 kb and was classified as belonging to class IV (**Table 2**), just as the chromosomal T4SS of *Xac* and *XccATCC* [78]. These findings are not unique to *Xam* CIO151, as one chromosomal and one plasmid copy of T4SS have been reported in *Xac* and *Xeu* as well [25], [47]. The difference in their organizations and the potential plasmidic origin of one of the two T4SS suggest that both secretion systems serve in distinct functions in *Xam* CIO151.

The versatile bacterial type VI secretion system (T6SS) is involved in interbacterial interactions, as well as symbiotic and pathogenic interactions with eukaryotic cells [80], [81] One copy of the T6SS with fifteen genes was detected in *Xam* CIO151, while two T6SS have been reported in *Xeu* and *X. oryzae* species [14].

The widely conserved *gum* cluster of xanthomonads [74], which is involved in xanthan exopolysaccharide (EPS) production [82], has been previously related to pathogenicity in *Xam* [19]. In *Xam* CIO151, the *gum* cluster is composed of the *gumABCDEFGHIJKLMNOP* genes (**Fig. 3B** **; Table S7**). It shows an organization comparable to that of other xanthomonads, including a tRNA gene adjacent to the cluster [74]. The G+C content of this region is 62.8%, two percentage points below the average value for the genome.

Lipopolysaccharides (LPS) are a characteristic element of bacterial outer membranes [83]. Interestingly, LPS molecules and components thereof have been classified as PAMPs (Pathogen Associated Molecular Patterns) in plant-pathogenic bacteria [84], [85]. Despite the fact that it is a conserved element in animal and plant pathogens, variations in the LPS cluster have been associated with virulence and host range [86]. The LPS gene cluster in *Xam* CIO151 consists of fifteen genes located in two consecutive scaffolds, with a total length of 17.2 kb. Ten of these genes are located in a region with atypical nucleotide composition (**Fig. S2**). The organization of the *Xam* CIO151 LPS locus is similar to that of *Xoo* strain BX08 and *Xac* strain 306 [87]. However, the LPS cluster of *Xam* CIO151 is distinct since it shares a fragment of an IS*1404* element with *Xoo* but is otherwise more similar in the order and number of genes with *Xac* (**Fig. S2**). Recently, very simlar LPS gene clusters have also been found in the banana pathogen *X. campestris* pv. *musacearum* strain NCPPB 4381 and in the pomegranate pathogen *X. axonopodis* pv. *punicae* strain LMG 859 [88], [89].

The Xa21 gene in rice recognizes an avirulence determinant present in *Xoo*, resulting in the elicitation of a strong defense response and suppression of pathogen growth. The presence and potential functionality of the genes *r*equired for *avrxa*21 activity (*rax*) was assessed in the genome of *Xam* CIO151. RaxH and RaxR, the two component regulatory system important for this activity, seems to be functional in *Xam* CIO151 (xanmn_chr10_0493 and xanmn_chr10_0494, respectively). RaxA and RaxC, a part of the Type I secretion system required for this activity also appear to be functional in *Xam* CIO151 (xanmn_chr10_0509 and xanmn_chr11_0184, respectively). However, the *raxB* gene (xanmn_chr10_0508), encoding the ATP-binding cassette transporter of the T1SS has a frameshift, resulting in a fragment encoding only 40% of the predicted functional protein, and suggesting a loss of function of this gene. This observation was confirmed in the 65 available genomes of *Xam*, demonstrating that it is a characteristic of the whole pathovar. In addition, a lack of function in some of these strains was previously suggested for the *raxST* genes encoding a tyrosine sulfotransferase [90]. These observations suggest that the AvrXa21 function might not be present in this pathovar.

### The set of type III effectors of *Xam* CIO151 is comparable in size with those of other xanthomonads

Effector proteins secreted by the T3SS play an important role in pathogenicity and virulence in Gram-negative bacteria. A total of twenty-eight CDS were identified as candidate effector genes (**Table S8**), based on homology searches against a database that includes type III effectors from different bacteria, including plant and animal pathogens (Rodríguez-R & Koebnik, unpublished data). Early stop codons were predicted for nine of these CDS, belonging to families XopAD, XopAG, XopF and XopP. In order to further confirm the pseudogenization of these fragments, we tested their expression in transcriptomic data of strains overexpressing the hrpX regulator (data not shown) using Bowtie aligner [91] followed by the Velvet assembler [92]. Analysis of transcriptomic data did not show any expression for xopP or xopAG, supporting their potential pseudogenization. In addition, the fragmentation of xopAG into two consecutive ORFs and the two adjacent copies of xopP were confirmed through comparisons with the sequences reported by Bart and collaborators [15]. The two copies of XopP are: one corresponding to the middle part of xopP (xanmn_chr10_0524) and the other copy fragmented in three consecutive ORFs (xanmn_chr10_0523, the N-terminal; xanmn_chr10_0522, the middle part; and xanmn_chr10_0521, the C-ter). We found expression of XopF in our transcriptome (data not shown) and the stop codon identified in the genome was confirmed. This suggests that the shorter version of xopF is still expressed.

The remaining nineteen genes (including xopF) belong to seventeen effector families (**Table 3**). Several fragmented sequences were inferred as part of candidate TAL effectors (AvrBs3/PthA family). Based on Southern blot analyses, strain CIO151 has two TAL effectors [26]. However, assembly of the genomic regions that encode TAL effectors is not accurate due to the presence of quasi-identical repeats in the TAL effector genes. Therefore, it is challenging to accurately determine the number and sequences of candidate TAL effector encoding genes from short read, draft genome assemblies [15].

**Table 3:** Comparison of putative effector proteins from *Xam* CIO151 and other members of the *Xanthomonas* genus.

Effector family	XamCIO151	Xeu^a^	XooMAFF^a^	Xac^a^	Xfa1^b^	XccATCC^a^
AvrBs1	-	1	-	-	-	1
AvrBs2	1	1	1	1	1	1
AvrBs3	2¤	-	17	4	2	-
XopB (HopD1)	-	1	-	-	2*	-
XopC1	-	1	-	-	-	-
XopC2	1+	2*	1	2*	-	-
XopD	-	1	-	-	-	1*
XopE1	1	1	-	1	1	-
XopE2	-	1	-	1	1	1
XopE3	-	-	-	1	1	-
XopE4	1	-	-	-	1	-
XopF1	1(F)	1	1	-	-	1*
XopF2	-	1	-	1*	1*	-
XopG	-	1	1*	-	-	1
XopH	-	1	-	-	-	1
XopI	-	1	1*	1	1	-
XopJ	-	2	-	-	1	1
XopK	1	1	1	1	1	1
XopL	1	1	1	1	1	1
XopN	1	1	1	1	1	1
XopO (HopK)	-	1	-	-	-	-
XopP	4* +	1	1	1	1	1
XopQ (HopQ1)	1	1	1	1	1	1
XopR	1	1	1	1	1	1
XopT	-	-	1	-	-	-
XopU	-	-	1	-	-	-
XopV	1	1	1	1	1	-
XopW	-	-	1	-	-	-
XopX	1	1	1	1	1	2
XopY	-	-	1	-	-	-
XopZ (HopAS1)	1	1	1	1	2*	1
XopAA	-	1	1	-	-	-
XopAB	-	-	1	-	-	-
XopAC	-	-	-	-	-	1
XopAD (SKWP)	3* +	3*	1	1	1	-
XopAE (HpaF)	1+	2*	1	1	1	-
XopAF (HopAF1)	-	-	-	-	1	-
XopAG (HopG1)	2*	-	-	-	1*	1
XopAH	-	-	-	-	-	1
XopAI	-	-	-	1	1	-
XopAJ	-	1	-	-	-	-
XopAK (HopK1)	1	1	-	1	1	-
XopAL	-	-	-	-	-	2
XopAM	-	-	-	-	-	1
XopAO^c^	2(+1)	-	-	-	-	-
Total&	19	27	37	22	22	21

Number in parentheses indicates number of genes in corresponding category.

a Data from effector families summarized by White et al. [13] and www.xanthomonas.org.

b Data reported by Moreira et al. [78].

c New effector family described by Potnis et al. [14].

* Possible pseudogenes.

+ Located in predicted genomic island.

(+1) One copy located in predicted genomic island.

F With a premature stop codon but may be still functional.

¤ Incomplete sequence due to repeats.

& Total number of potentially functional genes.

Because T3Es might define the host range and tissue specificity of xanthomonads, we compared the putative set of T3Es in *Xam* CIO151 with that of other members in the genus *Xanthomonas* (**Table 3**). This approach revealed a comparable number of effector families between *Xam* CIO151 and *Xeu*, *Xac*, *Xanthomonas fuscans* subsp. *aurantifolii* str. ICPB 11122 (*Xfa1*) [78] and *Xcc* species, each species having a specific T3E repertoire. In addition, the number of effector families, which appear to be non-functional due to the presence of early stop codons or frameshifts, does not significantly differ between *Xam* CIO151 and other sequenced xanthomonads (data not shown).

**Figure 4:**
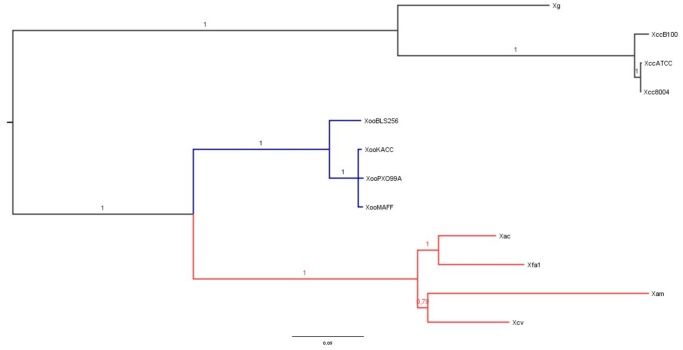
Phylogeny of conserved effectors in the genus *Xanthomonas*. Phylogenetic tree of concatenated conserved effector protein sequences of AvrBs2, XopK, XopL, XopN, XopQ XopR families and the Hpa1 protein, obtained with a Bayesian approach. Numbers on branches indicate Bayesian support values. Length of branches indicates the number of amino acid substitutions per site.

Genomics studies of Xanthomonad species have revealed a core set of conserved T3Es. This set includes AvrBs2, XopK, XopL, XopN, XopQ, XopR, XopX and XopZ, and was confirmed in *Xam* CIO151. In order to study the evolution of this set of proteins inside the genus *Xanthomonas*, a phylogenetic reconstruction was performed using these conserved effectors. XopZ and XopX were excluded due to the presence of more than one copy in some of the species. Hpa1 (XopA), which is a conserved type III-secreted protein of *Xanthomonas*, was also included in this analysis (**Fig. 4**). The phylogenetic tree suggests a common origin for the core effectors in the *X. axonopodis* clade consisting of *Xeu*, *Xac*, *Xfa1* and *Xam* CIO151. Our results are consistent with a previously published genomics scale phylogeny of the genus [93], suggesting that the evolution of these genes resembles species relationships within the genus and that they were probably present in the common ancestor of these taxa. In addition, the degree of conservation of this set of proteins within the genus *Xanthomonas* and the lack of co-location with recent HGT events in *Xam* CIO151 suggests that the acquisition of this set of effectors appears to be an ancient event.

Two genes (xanmn_chr06_5019 and xanmn_chr07_0047) showed high similarity, 95% and 81% at the amino acid level, to the recently reported effector family XopAO [14]. BLASTN and BLASTP searches in all available *Xanthomonas* genome sequences revealed that the XopAO effector family appears to be restricted to *X. gardneri* (*Xg*) and *Xam* CIO151, with *Xam* CIO151 possessing two members of this family. The *Xg xopAE* gene as well as one of the *Xam xopAE* genes are encoded in a region with atypical nucleotide composition (**Table 3**), which supports the possible acquisition of these genes through horizontal transfer events.

### Other determinants of interactions with plants in *Xam* CIO151

We also mined the genome sequence of *Xam* CIO151 for other pathogenicity-related determinants, such as chemotaxis, motility, the synthesis of second messengers and selected metabolic pathways, among others. This allowed us to generate a complete record of possible pathogenicity determinants. These analyses indicated the presence of genes related to chemotaxis, type IV pili (with corresponding genes distributed along the sequence of *Xam* CIO151), flagella (with a typical clustering of the corresponding genes), siderophore biosynthesis involved in iron uptake, and putative polyketide synthases (**Table 2**).

We also identified candidate adhesins in the genome sequence of *Xam* CIO151. Twenty four genes which are highly similar (identity ≥95% on more than 90% of the protein length) to *Xeu* or *Xac* type IV pilus (T4p) genes were identified in *Xam* CIO151 genome. The other T4p genes identified in *Xeu* and *Xac* genomes were not detected in *Xam* CIO151 or were too distantly related. Although no gene coding a pilin was identified among these genes, the presence of the genes encoding the sensor protein PilS, the ATPases PilB and PilT, may indicate that this T4p could be functional in *Xam* CIO151. Concerning the non-fimbrial adhesins, potential orthologs of autotransporters and filamentous hemagglutinins were identified in *Xam* CIO151 genome. One gene (chr06_0342) identified as *yapH2* and orthologous to XCV2103 harbors the specific domains of autotransporter adhesins. Two putative *xadA* genes were detected in *Xam* CIO151 genome (corresponding to locus tags chr_12_0029 and chr_12_0030 for the first one and to locus tags chr12_0032 and chr_12__0096 for the other). These putative *xadA* genes are split into two fragments in the genomic sequence. Regarding the *fhaB* family, *Xam* CIO151 has a gene similar to the filamentous hemagglutinin XAC4114, and two consecutive pseudogenes similar to the hemolysin activator protein XAC1815, which supports the duplication and decay observations from Mhedbi-Hajri and collaborators [94]. On the other hand, the *fhaC* gene, which is proposed as a helper in the translocation of FhaB adhesins, was not identified in *Xam* CIO151. The absence of this gene has also been reported in *Xeu* [94]. Due to this fact, the functionality of genes in the *fhaB* family is not clear in this *Xam* strain.

The role of cell wall degrading enzymes (CWDEs) *Xam* CIO151 pathogenicity has not been documented yet. However, they could be responsible for the ability of *Xam* to colonize xylem vessels, as it has recently been suggested for eukaryotic plant pathogens [95]. Based on homology analyses and manual genome annotation, we defined an inventory of 30 putative CWDEs and three related pseudogenes in *Xam* CIO151, including eight cellulases, two pectate lyases, five xylanases, two rhamnogalacturonases, one polygalacturonase, two endoglucanases, five beta-glucosidases and five alpha-glucuronidases (**Table S9**). All 30 candidate CWDEs have orthologous genes in other xanthomonads. Interestingly, the xylanase-encoding gene *xynB* seems to be absent, as observed for *Xv*, *Xg* and *Xcc* species [14]. Three consecutive cellullase genes were identified in a region with atypical composition. A gene encoding an alpha-glucuronidase of the GH67 family (*agu67*), which is present in all sequenced xanthomonads and which is clustered with xylanase genes in some xanthomonads, could be non-functional in *Xam* CIO151 due to a stop codon present in the middle of the coding sequence. This glucuronidase plays a joint role with xylanases during degradation of xylooligosaccharides in *Pseudomonas cellulosa* [96], and it has been proposed as a favorable element during the xylan degradation due to the limited ability of xylanases for cleaving uronic acid bones [97]. Hence, the absence of *xynB* suggests that xylan degradation is not highly efficient in *Xam* CIO151.

### Using the genome of *Xam* CIO151 as a source for easily accessible genotyping tools

**Figure 5:**
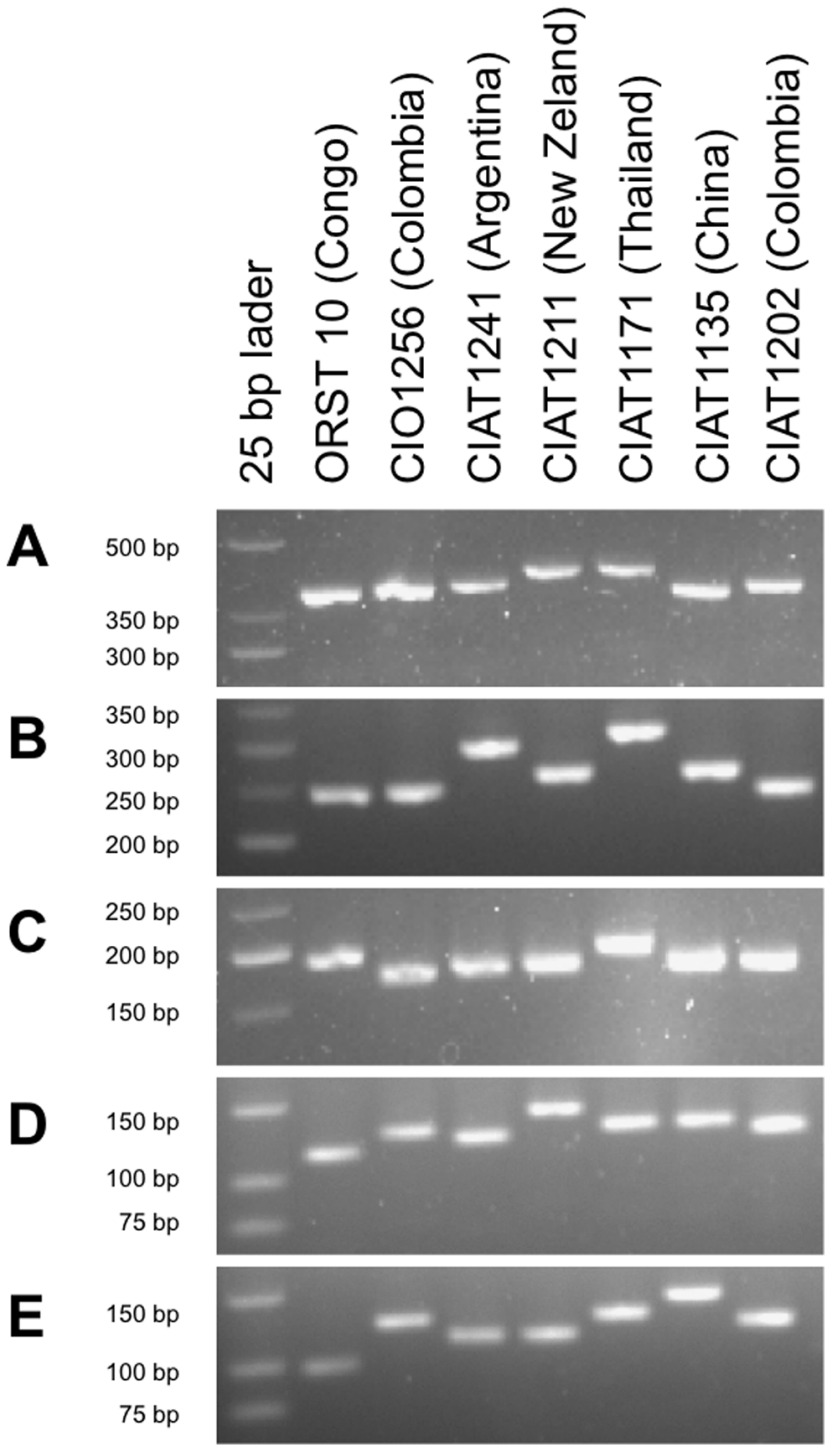
Molecular analysis of selected VNTR loci of *Xam*. PCR amplicons of VNTR loci of *Xam* were separated by agarose gel electrophoresis. A, XaG1_02 (362 bp); B, XaG1_29 (251 bp); C, XaG1_58 (192 bp); D, XaG2_50 (119 bp); E, XaG1_12 (111 bp). For comparison, expected sizes for *Xam* strain CIO151 are given in brackets.

Genomic resources offer the unique opportunity to develop genome-based molecular typing tools, such as MLVA schemes [34]. We used the genome sequence of *Xam* strain CIO151 to predict and evaluate VNTR loci. To develop a robust typing scheme, candidate loci were predicted for *Xam* strain CIO151 and three additional, phylogenetically close *Xanthomonas* strains, *X. axonopodis* pv. *citri* strain 306, *X. campestris* pv. *vesicatoria* strain 85-10, and *X. axonopodis* pv. *citrumelo* strain F1, using a web-based pipeline. Predicted loci for *Xam* that were also present and apparently polymorphic among these strains were targeted for primer design based on conserved sequence stretches within the flanking regions. A total of 14 primer pairs were generated and tested by PCR on 14 strains representative of the worldwide diversity of *Xam* (**Tables S10 and S11**). High-resolution agarose gel electrophoresis and/or DNA sequencing revealed that all 14 loci are polymorphic among this set of strains (**Fig. 5**, and data not shown).

To increase the number of loci, we also developed PCR primers for eight additional VNTR loci that appeared to be specific to *Xam* (**Table S10**). Since 65 draft genome sequences became available to us, we tested all 22 predicted loci on this valuable set of strains representing over 11 countries of origin belonging to three continents, and 70 years of collection [15]. We could detect and extract corresponding VNTR loci from 39 (XaG1_70) to 65 strains (average: 59 strains) (**Table S12**). Multiple alignments allowed us to estimate the exact number of repeats for each locus and the number of different patterns (haplotypes) per VNTR locus (**Tables 4** **and S12**).

**Table 4:** Characteristics of VNTR loci for 65 *Xam* draft genome sequences.

VNTR locus	Repeat unit size^1^	Number of repeats		Haplotypes			Samples with incomplete
		Min.^2^	Max.^3^	Number of samples^4^	Number of haplotypes^5^	HGDI score^6^	stuttered repeats
XaG1_73	6	3	16	60	12	0.903	
XaG1_67	6	9	22	51	12	0.900	
XaG1_02	7	8	20	48	12	0.894	
XaG1_29	7	10	22	48	10	0.854	
XaG1_71	6	3	12	57	9	0.778	23%
XaG2_50	6	5	12	61	7	0.774	
XaG1_70	7	10	15	39	6	0.773	8%
XaG1_72	6	4	11	59	8	0.710	
XaG1_65	6	7	12	65	6	0.670	
XaG1_58	6	3	9	65	7	0.658	
XaG1_12	7	4	9	63	5	0.635	35%
XaG2_52	13	4	10	52	7	0.839	
XaG2_37	24	1	2	64	2	0.476	
XaG2_55	12	2	5	65	3	0.146	
XaG1_105	8	3	11	64	8	0.694	
XaG1_101	7	4	6	63	3	0.618	24%
XaG1_108	6	3	4	65	2	0.306	
XaG1_110	21	2	4	59	3	0.523	
XaG2_109	26	2	3	56	2	0.486	
XaG2_106	22	2	3	65	2	0.170	
XaG2_116	22	1	2	65	2	0.089	
XaG2_117	25	1	2	64	2	0.031	

1 : Repeat unit sizes are given in bp.

2 and 3 : Minimal and maximal numbers of repeats (only those in integer numbers) are given.

4 Number of samples with a complete VNTR locus in the draft genome sequence is given.

5 Number of different VNTR patterns (haplotypes) is given.

6 Hunter-Gaston discriminatory index (HGDI) scores are given.

Discriminatory indices (HGDI) were calculated for all VNTR loci (**Table 4**). HGDI scores varied significantly from 0.031 for XaG2_117 to 0.903 for XaG1_73. As described previously [98], VNTR loci were classified into highly (>0.6), moderately (0.3 to 0.6), and poorly (<0.3) discriminating based on the HGDI scores. Of the 22 loci, 14 loci showed high discriminatory power. Four loci, XaG1_108, XaG1_110, XaG2_37 and XaG2_109, were found to be moderately discriminatory, and four loci, XaG2_55, XaG2_106, XaG2_116 and XaG2_117, had only poor discrimination. Four loci, XaG1_12, XaG1_70, XaG1_71 and XaG1_101, contained incomplete stuttered repeats which would pose problems in high-throughput analyses using multiplex PCR and amplicon analyses via capillary electrophoresis, resulting in uncertain calls of repeat numbers. These less useful loci, as well as the two loci with extremely poor discrimination (XaG2_116 and XaG2_117), were not considered for a broadly applicable MLVA scheme. Strikingly, combining the remaining 16 VNTR loci into an MLVA-16 scheme allowed resolution of almost all strains. Altogether, at least 57 haplotypes were observed among 65 strains, corresponding to 49 singletons and 8 doublets. Seven of the eight doublets include up to four VNTR loci for which the number of repeats could not be estimated for at least one strain due to the draft status of the genome sequence. In these cases, any number of repeats was taken into account when grouping strains into haplotypes. Hence, it is possible that these seven doublets could be experimentally resolved by the MLVA-16 scheme. Three doublets (IBSBF2345/IBSBF2346, IBSBF2672/IBSBF2673, UG24/UG27) originated from the same country and have been isolated in the same year (Brazil 2006, Brazil 2009, Uganda 2011). Two doublets (Xam669/IBSBF320, IBSBF2666/IBSBF2820) originated from the same country and have been isolated in two successive years (Brazil 1973/1974, Brazil 2009/2010). Another doublet (CFBP1851/CIO151) corresponds to two isolates from Colombia from years 1974 and 1995. The seventh doublet (UA556/IBSBF2818) was isolated in Colombia in 2009 and in Brazil in 2010. For the last doublet (ORST4/NCPPB1159), the origin of one strain, NCPPB1159, is unknown [15]. These results indicate that most if not all doublets likely correspond to truly related strains and do not result from homoplasy at certain VNTR loci. The MLVA-16 scheme translates into an excellent HGDI score of 0.996, thus demonstrating its suitability for typing of *Xam* strains.

## Discussion

This first expert-annotated high-quality draft genome sequence of *Xam* offers new insights into the genome structure of a bacterial plant pathogen affecting cassava crops and its candidate pathogenicity determinants. The comparison of the *Xam* CIO151 genome with other sequenced xanthomonads allowed us to propose a putative repertoire of 126 genes unique to *Xam* CIO151 that could play an important role in the cassava-*Xam* CIO151 interaction, possibly shaping properties such as host range and pathogenesis. Notably, among these genes, we identified both putative secreted proteins and membrane proteins, molecules widely recognized as bacterial pathogenicity determinants. Therefore, this group of genes constitutes an excellent candidate set for further research in order to understand the molecular basis underlying CBB and development of comprehensive control strategies for this disease.

The similarity of the *Xam* CIO151 genome sequence with those of *Xeu* and *Xac*, as well as the identification of clusters previously described as conserved in other xanthomonads, support the correct assembly of the genome sequence. Interestingly, in this and previous studies, phylogenomic relationships inferred from coding sequences (clusters of orthologous groups and conserved effectors) have placed *Xam* CIO151 within a monophyletic clade with *Xac* and *Xeu*, sister to the *Xoo* containing clade, and more distantly related to *Xcc* (Figure 3; [94]). Nonetheless, the similarity in the global genomic structure between *Xam* and that of other xanthomonads does not perfectly match such phylogenomic clustering. While the genomic structure of *Xam* CIO151 is more similar to that of *Xcc* and not to that of *Xoo* thus conflicting with the phylogenetic expectation This can be explained by the high number of recombination events reported in *Xoo* and the high number of insertion sequences [49], [99] Similar findings has been reported in the comparison between *Xac* and *Xoo* MAFF in global genome analysis [99]. These findings suggest that evolutionary processes leading to changes in genome organization are not entirely reflected in the conservation of genes.

Special features of *Xam* CIO151 such as a particular set of candidate T3Es and related pseudogenes were also identified. There were several T3Es present in *Xam* CIO151, which are not part of the conserved group among xanthomonads and which could be important determinants for its ability to colonize cassava. TAL effectors represent a challenge for second generation sequencing technologies at the stage of assembly of the central repeat region, and this was the case for *Xam* strain CIO151 as well. However, a member of this family has been demonstrated to be important for the ability of *Xam* to infect cassava [16]. The genome of *Xam* CIO151 encodes two TAL effectors and ongoing studies seek to determine their importance in the virulence of this strain.

As in other pathogenic bacteria, HGT events probably have contributed to the repertoire of T3Es in *Xam*. For instance, we detected XopAO in *Xam* CIO151, an effector that had only been identified in one more strain of xanthomonads, namely *X. gardneri*, where its functionality was confirmed and its origin from *Pseudomonas* via HGT was proposed [14]. Consistent with this speculation, our analyses support the hypothesis that *Xam* CIO151 acquired this effector via a recent HGT event. Additionally, we observed that the *xopP* pseudogenes and *xopC2* gene were located in a putative PAI (regions with atypical composition, mobile sequences and next to tRNA genes), whereas putative members of the XopAD and XopAE effector families were encoded by regions with atypical nucleotide composition. Altogether these observations suggest an important role for HGT events in recruiting a specific pathogenicity arsenal with probable consequences for microbial adaptations.

Four families of effectors may be pseudogenes in the genome sequence of *Xam* CIO151 (XopAD, XopAG, XopF and XopP). These effectors also seem inactive or absent in some but not all strains of *Xam* sequenced to-date [15] suggesting that their presence may be dispensable when infecting cassava plants. Consequently, when these T3Es were screened in a larger sample of *Xam* genomes, there was no correlation between the presence of a full-length version of these effectors and the levels of virulence [15]. It is possible that full-length functional versions of these proteins would induce an Effector-Triggered Immunity (ETI) in certain cultivars of cassava. No gene-for-gene interaction has been reported for this pathosystem. However, the quantitative resistance observed in certain cultivars might be the result of one or several of such interactions [7].

A Quantitative Trait Locus (QTL) for resistance to CBB in cassava co-segregates with a gene encoding a receptor-like kinase homologous to Xa21 from rice, a transmembrane protein containing an intracellular kinase domain [7], [8]. In the rice-*Xoo* system, rice immunity to *Xoo* is probably triggered after the recognition by the host Xa21 receptor of a bacterial secreted molecule [100]. The rax (required for AvrXa21 activity) genes in Xoo encode for a sulfotransferase, a two-component system and a transmembrane transport system. Comparisons with the *Xoo* sequence revealed frameshifts in the genes for the sulfotransferase [15] and the ABC transporter, which are both required for the AvrXa21 function. This finding suggests that *Xam* does not produce and secrete the molecule recognized by the rice Xa21 protein. Interestingly, a homolog of Xa21 co-segregates with a QTL for resistance to certain strains of *Xam* [7]. It is possible that this protein recognizes a molecule analogous to that expressed and secreted by Xoo. However, our findings indicate that secretion of this molecule is probably functionally different from that performed by *Xoo* in the *Xa21* system.

There are known variations in T2SS substrates among xanthomonads, even for conserved CWDEs in different species. For example, conserved substrates among several xanthomonads are apparently not secreted by the T2SSs in *Xeu* [101]. In addition, one of the T2SS clusters (*xcs*) is absent in *Xoo* [74] and, hence, the importance of this system for plant colonization is questionable. Taking this into account, future studies that evaluate the role of *xps* and *xcs* systems in *Xam* and their substrates are necessary to define their specific function, as well as to establish a curated set of CWDEs that are secreted via one or the other T2SS in *Xam*. In general, in terms of xylan degradation, the absence of the alpha-glucuronidase genes *agu67* and *xynB* is suprising, since a xylan is a prominent cell wall component in cassava (14.3%; [102]) and one would expect cassava pathogens to express a range of enzymes for its degradation. On the other hand, it is possible that *xynC*, a validated substrate of *xps* in *Xeu* [101], is sufficient for xylan degradation during infection in cassava, since it appears to be functional. These findings motivate further studies to determine the sufficiency of *xynC* in xylan degradation by *Xam* and its implications in pathogenicity and virulence of *Xam*.

The closer examination of the xanthomonadin cluster in *Xam* CIO151 sought to understand the atypical white coloration of this organism. Strikingly, in *Xam* CIO151 most xanthomonadin biosynthesis genes seem to be functional and only one gene encoding an acyl carrier protein dehydratase, which is necessary for xanthomonadin synthesis in *Xoo* strains [59], appears to be disrupted. This observation suggests that this gene may be responsible for the phenotypic shift in colouration observed in *Xam*. Interestingly, a BLAST search of this gene against the genome of *X. citri* pv. *mangiferaindicae* gave negative results (data not shown), suggesting that this might also cause the white phenotype of this bacterium. As xanthomonadin pigments have been implicated in epiphytic survival in other xanthomonads [57], their absence in *Xam* would imply the evolution of additional or novel characteristics involved in the survival of this pathogen during the epiphytic and early colonization phases [57], [103].

We found unique patterns in the LPS cluster of *Xam* CIO151. The organization of the lipopolysaccharide (LPS) biosynthesis cluster is highly variable within the genus *Xanthomonas* [87]. Our findings show that the organization of *Xam* CIO151 LPS cluster is similar to that of *Xac* and *XooBX08* suggesting that they may share a common ancestral LPS cluster. Simultaneously, the partial presence of LPS cluster in a region related to HGT is congruent with the hypothetical role of HGT as a source of diversity for this cluster, as has been proposed by Patil and Sonti [104].

Available genome sequences allowed us to develop and test a new molecular typing scheme. Sixteen out of twenty two tested VNTR loci were conserved in strains from three continents, Asia, Africa and South America and allowed to distinguish most of the *Xam* strains. Some of these loci might also be useful to type other, phylogenetically close pathovars of the same DNA-DNA hybridization group (group 9, [105]), including important pathogens of bean (*X. axonopodis* pv. *phaseoli*; group 9.4), alfalfae (*X. axonopodis* pv. *alfalfae*; group 9.2), soybean (*X. axonopodis* pv. *glycines*; group 9.5), lettuce (*X. axonopodis* pv. *vitians*; group 9.5), cotton (*X. axonopodis* pv. *malvacearum*; group 9.5), and cowpea (*X. axonopodis* pv. *vignicola*; group 9.6). The tremendous increase in whole genome sequencing will soon answer this question and lead to new, powerful typing tools for all economically important xanthomonads [6].

In conclusion, data mining based on the first annotated draft genome sequence of *Xam* CIO151 allowed, for the first time, the systematic cataloging of genes which might play a role in the interaction between *Xam* CIO151 and its host plant cassava, thus significantly increasing the sparse knowledge about molecular pathogenicity determinants of *Xam*. This new insight will pave the way for new approaches in the generation of durable resistant cassava varieties, thus leading to more efficient cassava disease management. New typing tools based on VNTR loci will allow an efficient and robust evaluation of population structures of *Xam* in different regions of the world and to implement comprehensive epidemiological surveillance, thus enabling better control of cassava bacterial blight.

## Materials and Methods

### *Xanthomonas* species abbreviation

*Xanthomonas albilineans* GPE PC7 (*Xal*)

*Xanthomonas citri* pv. *citri* str. 306 (*Xac*)

*Xanthomonas campestris* pv. *campestris* str. 8004 (*Xcc8004*)

*Xanthomonas campestris* pv. *campestris* str. ATCC 33913 (*XccATCC*)

*Xanthomonas campestris* pv. *campestris* str. B100 (*XccB100*)

*Xanthomonas campestris* pv. *vesicatoria* str. 85-10 (*Xeu*)

*Xanthomonas fuscans* subsp. *aurantifolii* str. ICPB 11122 (*Xfa1*)

*Xanthomonas oryzae* pv. *oryzae* KACC10331 (*XooKACC*)

*Xanthomonas oryzae* pv. *oryzae* PXO99^A^ (*XooPXO99A*)

*Xanthomonas oryzae* pv. *oryzae* MAFF 311018 (*XooMAFF*)

*Xanthomonas oryzae* pv. *oryzicola* BLS256 (*XocBLS256*)

*Xanthomonas perforans* 91–118 (*Xp*)

### Bacterial strain and DNA sequencing

The partial genome sequence was obtained with 454 technology (*shotgun* and paired-end tags) at Eurofins MWG Operon in USA (http://www.eurofins.com). The sequencing process was performed on total DNA (including plasmidic material) from *X. axonopodis* pv. *manihotis* strain CIO151 [9] extracted and purified as previously reported [27]. This strain was deposited as CFBP7661 in the French Collection of Plant-Associated Bacteria (CFBP).

### Genome assembly and structural and functional annotation

The genomic sequence of *Xam* CIO151 was assembled using Celera assembler [106]. A total of 40 scaffolds were produced. A manual inspection step allowed us to reduce the number of scaffolds to 36 by joining consecutive sequences, according to comparisons with *Xeu*, that have overlapping ends, or consecutive parts of conserved regions flanking rRNA operons. Total DNA that included possible extrachromosomal DNA was used in the sequencing process. For this reason, partial ordering of scaffolds was performed by alignments with the closely related genome sequence of *X. euvesicatoria* (*Xeu*) 85-10 (syn *X. campestris pv. vesicatoria*) [47]. This species was selected based on higher average nucleotide identity values [107] with *Xam* CIO151 [93]. Scaffolds that mapped to the *Xeu* genome were classified as the main chromosomal sequences (named xanmn_chr), scaffolds that mapped to plasmids in the genus Xanthomonas were classified as plasmidic (named xanmn_pla) and scaffolds that did not map against either of these two categories were classified as having an undertermined origin (named xanmn_unk). The circular representation of *Xam* CIO151 sequence was constructed using the Circos tool [108]. tRNAs search was performed using the tRNAscan-SE software [109]. Candidate rRNA operons were identified through a similarity analysis using sequences previously reported in other xanthomonads. The validation of the assembly was performed through an analysis of the GC content, genome length, search of genes already identified in *Xam* and comparisons to other *Xanthomonas* species using MUMmer 3 tool [110] and MAUVE [48].

The FrameD tool [111] was used for gene prediction because it increases the probability of identifying genes in sequences that might have problems with misread polynucleotides in some of the NGS technologies, including 454. It was trained with 100 conserved genes predicted with Glimmer3.02 [112]. Automatic and manual annotation was performed using iANT (integrated ANnotation Tool), as previously described [50]. The manual annotation was carried out by experts in subcategories of the Clusters of Orthologous Groups (COG) assigned by the automatic annotation. International experts on T3Es, adhesins, Two Component Systems, Mobile Elements, etc, collaborated in the manual annotation process. The manual annotation included addition and deletion of genes that were erroneously predicted by the automatic annotation system, labeling of genes that were splitted within scaffolds constructed with the Paired End data, correction of start codons taking into account homologous sequences and Ribosomal Binding Sites, specific assignation of names of genes where orthology had been positively tested. Identification of orthologous genes in other xanthomonads, protein domain descriptions, alignments and other characteristics were taken into account by annotators during the manual annotation process. The resulting information from assembly and annotation of the genome was deposited in Genbank as Bioproject number PRJNA202245 and in iANT at the website https://iant.toulouse.inra.fr/X.manihotis

### Identification of unique elements of *Xam* CIO151

Proteins in *Xam* CIO151 that do not have homologs in other xanthomonads were identified by OrthoMCL using the default parameters [113] as reported previously [50]. Based on the assumption that fragmented sequences could be classified as unique elements due to length differences, we repeated a BLAST [51] search of the previously identified proteins in the set proteins of sequenced xanthomonads using an e-value threshold of 1e^−10^. Additionally, genes with indeterminate nucleotide positions or genes that codified proteins with length lower than 50 aminoacids were dismissed. A BLAST search in the GENBANK database was conducted for the remaining sequences.

### Identification of putative horizontal transfer events

In order to identify regions with unusual composition, AlienHunter tool [114] was used. The predictions were evaluated though a search of tRNAs (including 5000 upstream and downstream), and mobile elements sequences available in the ACLAME database [115] (with an e-value threshold of 1e^−50^), on the putative regions predicted by AlienHunter. Identification of genes in *Xam* CIO151 that are shared with other xanthomonads was performed using OrthoMCL. Insertion sequences in *Xam* CIO151 were defined using data derived from the annotation process.

### Phylogenetic analysis

The phylogenetic reconstruction was performed using a concatenated sequence of conserved effector proteins: AvrBs2, XopK, XopL, XopN, XopQ, XopR and the Hpa1 regulon. Even though XopX and XopZ genes are conserved, they were excluded from this analysis because some species have multiple copies. Xanthomonads species included in the phylogenetic tree were: *Xam* CIO151, *XccATCC*, *Xcc8004*, *XccB100*, *XocBLS256*, *XooKACC*, *XooPXO99A*, *XooMAFF*, *Xeu*, *Xac*, *Xp* and *Xfa1*. A Bayesian approach using MrBayes software [116] with ten millions Markov Chain Monte Carlo, twenty percent of burnin, two runs and four chains, and a maximum likelihood approach using RaxML [117] with one thousand bootstrap replicates and an individual evolutionary model for each partition was used for the phylogenetic reconstruction. Protein alignments were produced with MUSCLE alignment tool [118]. Models of evolution were determined using the software Prottest [119], AIC criterion was used to determine the best-fitting model.

### Identification of clusters and pathogenicity determinants

Identification of *hrp*, *gum*, *rpf*, xanthomonadin and secretion systems clusters was performed through a manual annotation using available information for these clusters in other xanthomonads. The G+C content was computed for some of these clusters using the window-acgt tool from the glimmer3.02 package [112]. Elements related to effector proteins, toxins, cell wall degrading enzymes, chemotaxis and motility were identified using annotation data. The list of candidate effectors in sequenced xanthomonads was obtained from the website described in [13].

### Prediction of VNTR loci and design of PCR primers

The chromosomal sequences of *Xam* CIO151 and three phylogenetically close pathovars of *Xanthomonas* (*X. axonopodis* pv. *citri* strain 306 (GenBank accession number AE008923), *X. campestris* pv. *vesicatoria* strain 85-10 (GenBank accession number AM039952), and *X. axonopodis* pv. *citrumelo* strain F1 (GenBank accession number CP002914)) were scrutinized for the presence of the candidate VNTR loci using a web-based prediction pipeline (http://www.biopred.net/VNTR/; [25], [47], [120]. The Tandem Repeat Finder algorithm [121] was used and two sets of parameters were employed: (i) region length of 30 to 1000 bp, unit length of 5 to 9 bp, at least 6 copies and an identity of a least 80% between adjacent repeats, and (ii) region length of 20 to 1000 bp, unit length of 10 to 26 bp, at least 2 copies and an identity of a least 80% between adjacent repeats. Predicted VNTR loci were grouped according to their shared 500-bp flanking regions. Loci that were predicted for *Xam* and present in at least two other strains were further evaluated.

Homologous 500-bp regions next to the predicted VNTR loci were extracted from the chromosomal sequences and aligned using MUSCLE (http://www.ebi.ac.uk/Tools/msa/muscle/) [118]. PCR primers matching to conserved segments were designed using the Finnzymes website (http://www.finnzymes.fi/tm_determination.html; **Table S10**). Designed primer sequences were queried against the chromosomal sequences using the MFEprimer website (http://biocompute.bmi.ac.cn/MFEprimer/; [122]) to confirm that the primer pairs will only amplify one locus per genome.

### PCR amplification, agarose gel electrophoresis and DNA sequencing

Candidate polymorphic loci were tested on a set of at least 14 strains of *Xam* representing worldwide diversity (**Table S11**). PCR amplifications were performed using genomic DNA of *Xam* strains as template DNA. Each PCR reaction was carried out in a final volume of 25 µl and contained 10–50 ng genomic DNA, 2.5 mM MgCl_2_, 40 nM PCR primers, 2 mM dNTP and 1 unit of *Taq* DNA polymerase (Promega, USA). All reactions were run for 35 cycles, each consisting of 20 sec at 95°C, 30 sec at 52–58°C (depending on the primer pair), and 30–60 sec at 72°C, with an initial denaturation step of 3 min at 95°C and a final extension step of 10 min at 72°C. PCR products were separated on agarose gels and, if required, sent for custom DNA sequencing (Beckman Coulter Genomics, UK).

### Computational analysis of VNTR loci among 65 *Xam* draft genome sequences

All VNTR primer pairs (**Table S10**) were also tested on 65 recently released draft genome sequences of *Xam* [15] using in-house developed scripts. Numbers of complete repeats were determined from multiple alignments of all draft genome sequences. The allelic profile of a given strain was defined as the repeat numbers at each VNTR locus. The discriminatory power of all VNTR loci were calculated using the Hunter-Gaston discriminatory index (HGDI), using the following formula:
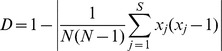
where D is the numerical index of discriminatory power, N is the total number of strains in the typing scheme, s is the total number of different strain types, and xj is the number of strains belonging to the j^th^ type [123].

## Supporting Information

Figure S1 Alignment of putative chromosomal scaffolds of *Xam* CIO151 and *Xeu*, *Xac*, *Xcc8004* and *XooPXO99A* chromosomes using MAUVE software. **A**. Alignment between *Xam* CIO151 and *Xeu*, **B.** Alignment between *Xam* and *Xac*, **C.** Alignment between *Xcc8004* and *Xam* CIO151, **D.** Alignment between *XooPXO99A* and *Xam* CIO151. Vertical red lines in *Xam* CIO151 indicate the scaffolds.
(TIFF)

Figure S2 Comparison of lipopolysaccharide gene clusters of *Xam*, *XooBX08* and *Xac*. Homologous genes are represented by the same color. Dotted lines in *Xam* indicates the distribution of the cluster on two consecutives scaffolds.
(TIFF)

Table S1 Predicted proteins unique to *Xam*.
(DOC)

Table S2 Characteristics of chromosomal regions predicted with atypical nucleotide composition.
(DOC)

Table S3 Potential pathogenicity associated islands (PAIs) in *Xam*.
(DOC)

Table S4 *rpf* gene cluster of *Xam* CIO151.
(DOCX)

Table S5 Type II secretion systems in *Xam* CIO151.
(DOC)

Table S6 Type III secretion system (*hrp* cluster) in *Xam* CIO151.
(DOCX)

Table S7 Xanthan *gum* gene cluster in *Xam* CIO151.
(DOC)

Table S8 CDS of putative effector proteins detected in the genome of *Xam* CIO151.
(DOCX)

Table S9 Putative set of cell wall-degrading enzymes present in *Xam* CIO151.
(DOCX)

Table S10 Oligonucleotide primers, PCR conditions, and characteristics of VNTRs analyzed in this study.
(DOCX)

Table S11 List of *Xam* strains used to evaluate VNTR primers.
(DOCX)

Table S12 Presence and characteristics of VNTR markers in 65 genome sequences of *Xam* worldwide.
(PDF)

## References

[1] Leyns F, Cleene M, Swings JG, Ley J (1984) The host range of the genus *Xanthomonas* . Bot Rev 50: 308–356.

[2] Hayward A, Swings J, Civerolo E (1993) The hosts of *Xanthomonas* In: Swings J, Civerolo E, editors. *Xanthomonas*. London, United Kingdom: Chapman & Hall. pp. 1–119.

[3] Lozano JC (1986) Cassava bacterial blight: a manageable disease. Plant Dis 70: 1089–1093.

[4] Lozano J, Sequeira L (1973) Bacterial blight of cassava in Colombia: epidemiology and control. Phytopathology 64: 83–88.

[5] Boher B, Verdier V (1994) Cassava bacterial blight in Africa: the state of knowledge and implications for designing control strategies. Afr Crop Sci J 2: 505–509.

[6] Mansfield J, Genin S, Magori S, Citovsky V, Sriariyanum M, et al (2012) Top 10 plant pathogenic bacteria in molecular plant pathology. Mol Plant Pathol 13: 614–629. 22672649 10.1111/j.1364-3703.2012.00804.x PMC6638704

[7] Lopez C, Bernal A (2012) Cassava Bacterial Blight: Using Genomics for the Elucidation and Management of an Old Problem. Tropical Plant Biology 5: 117–126.

[8] Jorge V, Fregene MA, Duque MC, Bonierbale MW, Tohme J, et al (2000) Genetic mapping of resistance to bacterial blight disease in cassava (*Manihot esculenta* Crantz). Theor Appl Genet 101: 865–872.

[9] Restrepo S, Duque MC, Verdier V (2000) Characterization of pathotypes among isolates of *Xanthomonas axonopodis* pv. *manihotis* in Colombia. Plant Pathol 49: 680–687.

[10] Buttner D, Bonas U (2010) Regulation and secretion of *Xanthomonas* virulence factors. FEMS Microbiol Rev 34: 107–133. 19925633 10.1111/j.1574-6976.2009.00192.x

[11] Hajri A, Brin C, Hunault G, Lardeux F, Lemaire C, et al (2009) A «repertoire for repertoire» hypothesis: Repertoires of type three effectors are candidate determinants of host specificity in *Xanthomonas* . PLoS One 4: e6632. 19680562 10.1371/journal.pone.0006632 PMC2722093

[12] Koebnik R, Lindeberg M (2011) Comparative Genomics and Evolution of Bacterial Type III Effectors. In: Martin F, Kamoun S, editors. Effectors in Plant–Microbe Interactions: Wiley-Blackwell. pp. 53–76.

[13] White FF, Potnis N, Jones JB, Koebnik R (2009) The type III effectors of *Xanthomonas* . Mol Plant Pathol 10: 749–766. 19849782 10.1111/j.1364-3703.2009.00590.x PMC6640274

[14] Potnis N, Krasileva K, Chow V, Almeida NF, Patil PB, et al (2011) Comparative genomics reveals diversity among xanthomonads infecting tomato and pepper. BMC Genomics 12: 146. 21396108 10.1186/1471-2164-12-146 PMC3071791

[15] Bart R, Cohn M, Kassen A, McCallum EJ, Shybut M, et al (2012) High-throughput genomic sequencing of cassava bacterial blight strains identifies conserved effectors to target for durable resistance. Proc Natl Acad Sci U S A 109: E1972–1979. 22699502 10.1073/pnas.1208003109 PMC3396514

[16] Castiblanco LF, Gil J, Rojas A, Osorio D, Gutierrez S, et al (2013) TALE1 from *Xanthomonas axonopodis* pv. *manihotis* acts as a transcriptional activator in plant cells and is important for pathogenicity in cassava plants. Mol Plant Pathol 14: 84–95. 22947214 10.1111/j.1364-3703.2012.00830.x PMC6638846

[17] Boher B, Kpemoua K, Nicole M, Luisetti J, Geiger JP (1995) Ultrastructure of interactions between cassava and *Xanthomonas campestris* pv. *manihotis*: Cytochemistry of cellulose and pectin degradation in a susceptible cultivar. Phytopathology 85: 777–788.

[18] Boher B, Nicole M, Potin M, Geiger JP (1997) Extracellular polysaccharides from *Xanthomonas axonopodis* pv. *manihotis* interact with cassava cell walls during pathogenesis. Mol Plant Microbe Interact 10: 803–811. 9304855 10.1094/MPMI.1997.10.7.803

[19] Kemp BP, Horne J, Bryant A, Cooper RM (2004) *Xanthomonas axonopodis* pv. *manihotis* gumD gene is essential for EPS production and pathogenicity and enhances epiphytic survival on cassava (Manihot esculenta). Physiol Mol Plant Pathol 64: 209–218.

[20] Ochman H, Lawrence JG, Groisman EA (2000) Lateral gene transfer and the nature of bacterial innovation. Nature 405: 299–304. 10830951 10.1038/35012500

[21] Hentschel U, Hacker J (2001) Pathogenicity islands: the tip of the iceberg. Microbes Infect 3: 545–548. 11418328 10.1016/s1286-4579(01)01410-1

[22] Vernikos GS, Thomson NR, Parkhill J (2007) Genetic flux over time in the *Salmonella* lineage. Genome Biol 8: R100. 17547764 10.1186/gb-2007-8-6-r100 PMC2394748

[23] Lima WC, Paquola AC, Varani AM, Van Sluys MA, Menck CF (2008) Laterally transferred genomic islands in *Xanthomonadales* related to pathogenicity and primary metabolism. FEMS Microbiol Lett 281: 87–97. 18318843 10.1111/j.1574-6968.2008.01083.x

[24] Noel L, Thieme F, Nennstiel D, Bonas U (2002) Two novel type III-secreted proteins of *Xanthomonas campestris* pv. *vesicatoria* are encoded within the hrp pathogenicity island. J Bacteriol 184: 1340–1348. 11844763 10.1128/JB.184.5.1340-1348.2002 PMC134860

[25] da Silva AC, Ferro JA, Reinach FC, Farah CS, Furlan LR, et al (2002) Comparison of the genomes of two *Xanthomonas* pathogens with differing host specificities. Nature 417: 459–463. 12024217 10.1038/417459a

[26] Verdier V, Boher B, Maraite H, Geiger JP (1994) Pathological and Molecular Characterization of *Xanthomonas campestris* Strains Causing Diseases of Cassava (*Manihot esculenta*). Appl Environ Microbiol 60: 4478–4486. 16349463 10.1128/aem.60.12.4478-4486.1994 PMC202008

[27] Restrepo S, Verdier V (1997) Geographical Differentiation of the Population of *Xanthomonas axonopodis* pv. *manihotis* in Colombia. Appl Environ Microbiol 63: 4427–4434. 16535731 10.1128/aem.63.11.4427-4434.1997 PMC1389287

[28] Restrepo S, Duque M, Tohme J, Verdier V (1999) AFLP fingerprinting: an efficient technique for detecting genetic variation of *Xanthomonas axonopodis* pv. *manihotis* . Microbiology 145 (Pt 1) 107–114. 10206688 10.1099/13500872-145-1-107

[29] Restrepo S, Velez CM, Verdier V (2000) Measuring the Genetic Diversity of *Xanthomonas axonopodis* pv. *manihotis* Within Different Fields in Colombia. Phytopathology 90: 683–690. 18944486 10.1094/PHYTO.2000.90.7.683

[30] Gonzalez C, Restrepo S, Tohme J, Verdier V (2002) Characterization of pathogenic and nonpathogenic strains of *Xanthomonas axonopodis* pv. *manihotis* by PCR-based DNA fingerprinting techniques. FEMS Microbiol Lett 215: 23–31. 12393196 10.1111/j.1574-6968.2002.tb11365.x

[31] Restrepo S, Velez CM, Duque MC, Verdier V (2004) Genetic structure and population dynamics of *Xanthomonas axonopodis* pv. *manihotis* in Colombia from 1995 to 1999. Appl Environ Microbiol 70: 255–261. 14711649 10.1128/AEM.70.1.255-261.2004 PMC321237

[32] van Belkum A (2007) Tracing isolates of bacterial species by multilocus variable number of tandem repeat analysis (MLVA). FEMS Immunol Med Microbiol 49: 22–27. 17266711 10.1111/j.1574-695X.2006.00173.x

[33] Vergnaud G, Pourcel C (2009) Multiple locus variable number of tandem repeats analysis. Methods Mol Biol 551: 141–158. 19521873 10.1007/978-1-60327-999-4_12

[34] Li W, Raoult D, Fournier PE (2009) Bacterial strain typing in the genomic era. FEMS Microbiol Rev 33: 892–916. 19453749 10.1111/j.1574-6976.2009.00182.x

[35] Lindstedt BA (2005) Multiple-locus variable number tandem repeats analysis for genetic fingerprinting of pathogenic bacteria. Electrophoresis 26: 2567–2582. 15937984 10.1002/elps.200500096

[36] Coletta-Filho HD, Takita MA, de Souza AA, Aguilar-Vildoso CI, Machado MA (2001) Differentiation of strains of *Xylella fastidiosa* by a variable number of tandem repeat analysis. Appl Environ Microbiol 67: 4091–4095. 11526010 10.1128/AEM.67.9.4091-4095.2001 PMC93134

[37] Ngoc LB, Verniere C, Vital K, Guerin F, Gagnevin L, et al (2009) Development of 14 minisatellite markers for the citrus canker bacterium, *Xanthomonas citri* pv. citri. Mol Ecol Resour 9: 125–127. 21564579 10.1111/j.1755-0998.2008.02242.x

[38] Gironde S, Manceau C (2012) Housekeeping gene sequencing and multilocus variable-number tandem-repeat analysis to identify subpopulations within *Pseudomonas syringae* pv. *maculicola* and *Pseudomonas syringae* pv. *tomato* that correlate with host specificity. Appl Environ Microbiol 78: 3266–3279. 22389364 10.1128/AEM.06655-11 PMC3346470

[39] Katoh H, Subandiyah S, Tomimura K, Okuda M, Su HJ, et al (2011) Differentiation of “*Candidatus* Liberibacter asiaticus” isolates by variable-number tandem-repeat analysis. Appl Environ Microbiol 77: 1910–1917. 21239554 10.1128/AEM.01571-10 PMC3067300

[40] Pruvost O, Verniere C, Vital K, Guerin F, Jouen E, et al (2011) Insertion sequence- and tandem repeat-based genotyping techniques for *Xanthomonas citri* pv. *mangiferaeindicae* . Phytopathology 101: 887–893. 21323466 10.1094/PHYTO-11-10-0304

[41] Zhao S, Poulin L, Rodriguez RL, Serna NF, Liu SY, et al (2012) Development of a variable number of tandem repeats typing scheme for the bacterial rice pathogen *Xanthomonas oryzae* pv. *oryzicola* . Phytopathology 102: 948–956. 22957820 10.1094/PHYTO-04-12-0078-R

[42] Lopez CE, Quesada-Ocampo LM, Bohorquez A, Duque MC, Vargas J, et al (2007) Mapping EST-derived SSRs and ESTs involved in resistance to bacterial blight in *Manihot esculenta* . Genome 50: 1078–1088. 18059536 10.1139/G07-087

[43] Lopez C, Soto M, Restrepo S, Piegu B, Cooke R, et al (2005) Gene expression profile in response to *Xanthomonas axonopodis* pv. *manihotis* infection in cassava using a cDNA microarray. Plant Mol Biol 57: 393–410. 15830129 10.1007/s11103-004-7819-3

[44] Santaella M, Suarez E, Lopez C, Gonzalez C, Mosquera G, et al (2004) Identification of genes in cassava that are differentially expressed during infection with *Xanthomonas axonopodis* pv. *manihotis* . Mol Plant Pathol 5: 549–558. 20565629 10.1111/j.1364-3703.2004.00254.x

[45] Ryan RP, Vorholter FJ, Potnis N, Jones JB, Van Sluys MA, et al (2011) Pathogenomics of *Xanthomonas*: understanding bacterium-plant interactions. Nat Rev Microbiol 9: 344–355. 21478901 10.1038/nrmicro2558

[46] Lobry JR (1996) Asymmetric substitution patterns in the two DNA strands of bacteria. Mol Biol Evol 13: 660–665. 8676740 10.1093/oxfordjournals.molbev.a025626

[47] Thieme F, Koebnik R, Bekel T, Berger C, Boch J, et al (2005) Insights into genome plasticity and pathogenicity of the plant pathogenic bacterium *Xanthomonas campestris* pv. *vesicatoria* revealed by the complete genome sequence. J Bacteriol 187: 7254–7266. 16237009 10.1128/JB.187.21.7254-7266.2005 PMC1272972

[48] Darling AE, Mau B, Perna NT (2010) progressiveMauve: multiple genome alignment with gene gain, loss and rearrangement. PLoS One 5: e11147. 20593022 10.1371/journal.pone.0011147 PMC2892488

[49] Salzberg SL, Sommer DD, Schatz MC, Phillippy AM, Rabinowicz PD, et al (2008) Genome sequence and rapid evolution of the rice pathogen *Xanthomonas oryzae* pv. *oryzae* PXO99A. BMC Genomics 9: 204. 18452608 10.1186/1471-2164-9-204 PMC2432079

[50] Pieretti I, Royer M, Barbe V, Carrere S, Koebnik R, et al (2009) The complete genome sequence of *Xanthomonas albilineans* provides new insights into the reductive genome evolution of the xylem-limited *Xanthomonadaceae* . BMC Genomics 10: 616. 20017926 10.1186/1471-2164-10-616 PMC2810307

[51] Altschul SF, Madden TL, Schaffer AA, Zhang J, Zhang Z, et al (1997) Gapped BLAST and PSI-BLAST: a new generation of protein database search programs. Nucleic Acids Res 25: 3389–3402. 9254694 10.1093/nar/25.17.3389 PMC146917

[52] Lawrence JG, Hendrickson H (2003) Lateral gene transfer: when will adolescence end? Mol Microbiol 50: 739–749. 14617137 10.1046/j.1365-2958.2003.03778.x

[53] Lerat E, Daubin V, Ochman H, Moran NA (2005) Evolutionary origins of genomic repertoires in bacteria. PLoS Biol 3: e130. 15799709 10.1371/journal.pbio.0030130 PMC1073693

[54] Thebault P, Servant F, Schiex D, Kahn D, Gouzy J. L'environnement iANT: integrated annotation tool. In: Sagot OGaMF, editor; 2000; Montpellier, France. Springer-Verlag. pp. 361–365.

[55] Hacker J, Blum-Oehler G, Muhldorfer I, Tschape H (1997) Pathogenicity islands of virulent bacteria: structure, function and impact on microbial evolution. Mol Microbiol 23: 1089–1097. 9106201 10.1046/j.1365-2958.1997.3101672.x

[56] Starr MP (1981) The prokaryotes. In: Starr MP, Stolp H, Truper HG, Balows A, Schlegel HG, editors. The prokaryotes. Berlin: Springer-Verlag. pp. 742–763.

[57] Poplawsky AR, Urban SC, Chun W (2000) Biological role of xanthomonadin pigments in *Xanthomonas campestris* pv. *campestris* . Appl Environ Microbiol 66: 5123–5127. 11097878 10.1128/aem.66.12.5123-5127.2000 PMC92432

[58] Poplawsky AR, Chun W (1997) pigB determines a diffusible factor needed for extracellular polysaccharide slime and xanthomonadin production in *Xanthomonas campestris* pv. *campestris* . J Bacteriol 179: 439–444. 8990296 10.1128/jb.179.2.439-444.1997 PMC178714

[59] Goel AK, Rajagopal L, Nagesh N, Sonti RV (2002) Genetic locus encoding functions involved in biosynthesis and outer membrane localization of xanthomonadin in *Xanthomonas oryzae* pv. *oryzae* . J Bacteriol 184: 3539–3548. 12057948 10.1128/JB.184.13.3539-3548.2002 PMC135150

[60] Von Bodman SB, Bauer WD, Coplin DL (2003) Quorum sensing in plant-pathogenic bacteria. Annu Rev Phytopathol 41: 455–482. 12730390 10.1146/annurev.phyto.41.052002.095652

[61] Dow M (2008) Diversification of the function of cell-to-cell signaling in regulation of virulence within plant pathogenic xanthomonads. Sci Signal 1: pe23. 18506032 10.1126/stke.121pe23

[62] He YW, Zhang LH (2008) Quorum sensing and virulence regulation in *Xanthomonas campestris* . FEMS Microbiol Rev 32: 842–857. 18557946 10.1111/j.1574-6976.2008.00120.x

[63] Wang L, Makino S, Subedee A, Bogdanove AJ (2007) Novel candidate virulence factors in rice pathogen *Xanthomonas oryzae* pv. *oryzicola* as revealed by mutational analysis. Appl Environ Microbiol 73: 8023–8027. 17981946 10.1128/AEM.01414-07 PMC2168139

[64] Barber CE, Tang JL, Feng JX, Pan MQ, Wilson TJ, et al (1997) A novel regulatory system required for pathogenicity of *Xanthomonas campestris* is mediated by a small diffusible signal molecule. Mol Microbiol 24: 555–566. 9179849 10.1046/j.1365-2958.1997.3721736.x

[65] Chatterjee S, Sonti RV (2002) rpfF mutants of *Xanthomonas oryzae* pv. *oryzae* are deficient for virulence and growth under low iron conditions. Mol Plant Microbe Interact 15: 463–471. 12036277 10.1094/MPMI.2002.15.5.463

[66] Dow JM, Crossman L, Findlay K, He YQ, Feng JX, et al (2003) Biofilm dispersal in *Xanthomonas campestris* is controlled by cell-cell signaling and is required for full virulence to plants. Proc Natl Acad Sci U S A 100: 10995–11000. 12960398 10.1073/pnas.1833360100 PMC196915

[67] Siciliano F, Torres P, Sendí­n L, Bermejo C, Filippone P, et al (2006) Analysis of the molecular basis of *Xanthomonas axonopodis* pv. *citri* pathogenesis in *Citrus* limon. Electron J Biotechn 9: 0–0.

[68] Slater H, Alvarez-Morales A, Barber CE, Daniels MJ, Dow JM (2000) A two-component system involving an HD-GYP domain protein links cell-cell signalling to pathogenicity gene expression in *Xanthomonas campestris* . Mol Microbiol 38: 986–1003. 11123673 10.1046/j.1365-2958.2000.02196.x

[69] Ryan RP, McCarthy Y, Andrade M, Farah CS, Armitage JP, et al (2010) Cell-cell signal-dependent dynamic interactions between HD-GYP and GGDEF domain proteins mediate virulence in *Xanthomonas campestris* . Proc Natl Acad Sci U S A 107: 5989–5994. 20231439 10.1073/pnas.0912839107 PMC2851925

[70] Ryan RP, Fouhy Y, Lucey JF, Crossman LC, Spiro S, et al (2006) Cell-cell signaling in *Xanthomonas campestris* involves an HD-GYP domain protein that functions in cyclic di-GMP turnover. Proc Natl Acad Sci U S A 103: 6712–6717. 16611728 10.1073/pnas.0600345103 PMC1458946

[71] Dow JM, Scofield G, Trafford K, Turner PC, Daniels MJ (1987) A gene cluster in *Xanthomonas campestris* pv. *campestris* required for pathogenicity controls the excretion of polygalacturonate lyase and other enzymes. Physiol Mol Plant Path 31: 261–271.

[72] Barras F, van Gijsegem F, Chatterjee AK (1994) Extracellular enzymes and pathogenesis of soft-rot *Erwinia* . Annu Rev Phytopathol 32: 201–234.

[73] Kang Y, Huang J, Mao G, He LY, Schell MA (1994) Dramatically reduced virulence of mutants of *Pseudomonas solanacearum* defective in export of extracellular proteins across the outer membrane. MPMI 7: 370–377.

[74] Lu H, Patil P, Van Sluys MA, White FF, Ryan RP, et al (2008) Acquisition and evolution of plant pathogenesis–associated gene clusters and candidate determinants of tissue-specificity in *Xanthomonas* . PLoS One 3: e3828. 19043590 10.1371/journal.pone.0003828 PMC2585010

[75] Cornelis GR, Van Gijsegem F (2000) Assembly and function of type III secretory systems. Annu Rev Microbiol 54: 735–774. 11018143 10.1146/annurev.micro.54.1.735

[76] Cascales E, Christie PJ (2003) The versatile bacterial type IV secretion systems. Nat Rev Microbiol 1: 137–149. 15035043 10.1038/nrmicro753 PMC3873781

[77] Hubber A, Vergunst AC, Sullivan JT, Hooykaas PJ, Ronson CW (2004) Symbiotic phenotypes and translocated effector proteins of the *Mesorhizobium loti* strain R7A VirB/D4 type IV secretion system. Mol Microbiol 54: 561–574. 15469524 10.1111/j.1365-2958.2004.04292.x

[78] Moreira LM, Almeida NF Jr, Potnis N, Digiampietri LA, Adi SS, et al (2010) Novel insights into the genomic basis of citrus canker based on the genome sequences of two strains of *Xanthomonas fuscans* subsp. *aurantifolii* . BMC Genomics 11: 238. 20388224 10.1186/1471-2164-11-238 PMC2883993

[79] Segal G, Russo JJ, Shuman HA (1999) Relationships between a new type IV secretion system and the icm/dot virulence system of *Legionella pneumophila* . Mol Microbiol 34: 799–809. 10564519 10.1046/j.1365-2958.1999.01642.x

[80] Records AR (2011) The type VI secretion system: a multipurpose delivery system with a phage-like machinery. Mol Plant Microbe Interact 24: 751–757. 21361789 10.1094/MPMI-11-10-0262

[81] Russell AB, LeRoux M, Hathazi K, Agnello DM, Ishikawa T, et al (2013) Diverse type VI secretion phospholipases are functionally plastic antibacterial effectors. Nature 496: 508–512. 23552891 10.1038/nature12074 PMC3652678

[82] Katzen F, Ferreiro DU, Oddo CG, Ielmini MV, Becker A, et al (1998) *Xanthomonas campestris* pv. *campestris* gum mutants: effects on xanthan biosynthesis and plant virulence. J Bacteriol 180: 1607–1617. 9537354 10.1128/jb.180.7.1607-1617.1998 PMC107069

[83] Osborn MJ, Rosen SM, Rothfield L, Zeleznick LD, Horecker BL (1964) Lipopolysaccharide of the gram-negative cell wall. Science 145: 783. 14163315 10.1126/science.145.3634.783

[84] Medzhitov R, Janeway Jr CA (1997) Innate Immunity: Minireview The Virtues of a Nonclonal System of Recognition. Cell 91: 295–298. 9363937 10.1016/s0092-8674(00)80412-2

[85] Casabuono A, Petrocelli S, Ottado J, Orellano EG, Couto AS (2011) Structural analysis and involvement in plant innate immunity of *Xanthomonas axonopodis* pv. *citri* lipopolysaccharide. J Biol Chem 286: 25628–25643. 21596742 10.1074/jbc.M110.186049 PMC3138316

[86] Mooi FR, Bik EM (1997) The evolution of epidemic *Vibrio cholerae* strains. Trends Microbiol 5: 161–165. 9141191 10.1016/S0966-842X(96)10086-X

[87] Patil PB, Bogdanove AJ, Sonti RV (2007) The role of horizontal transfer in the evolution of a highly variable lipopolysaccharide biosynthesis locus in xanthomonads that infect rice, citrus and crucifers. BMC Evol Biol 7: 243. 18053269 10.1186/1471-2148-7-243 PMC2238763

[88] Studholme DJ, Kemen E, MacLean D, Schornack S, Aritua V, et al (2010) Genome-wide sequencing data reveals virulence factors implicated in banana *Xanthomonas* wilt. FEMS Microbiol Lett 310: 182–192. 20695894 10.1111/j.1574-6968.2010.02065.x

[89] Sharma V, Midha S, Ranjan M, Pinnaka AK, Patil PB (2012) Genome sequence of *Xanthomonas axonopodis* pv. *punicae* strain LMG 859. J Bacteriol 194: 2395. 22493202 10.1128/JB.00181-12 PMC3347085

[90] Han SW, Lee SW, Bahar O, Schwessinger B, Robinson MR, et al (2012) Tyrosine sulfation in a Gram-negative bacterium. Nat Commun 3: 1153. 23093190 10.1038/ncomms2157 PMC4305400

[91] Langmead B, Trapnell C, Pop M, Salzberg SL (2009) Ultrafast and memory-efficient alignment of short DNA sequences to the human genome. Genome Biol 10: R25. 19261174 10.1186/gb-2009-10-3-r25 PMC2690996

[92] Zerbino DR, Birney E (2008) Velvet: algorithms for de novo short read assembly using de Bruijn graphs. Genome research 18: 821–829. 18349386 10.1101/gr.074492.107 PMC2336801

[93] Rodriguez RL, Grajales A, Arrieta-Ortiz ML, Salazar C, Restrepo S, et al (2012) Genomes-based phylogeny of the genus *Xanthomonas* . BMC Microbiol 12: 43. 22443110 10.1186/1471-2180-12-43 PMC3359215

[94] Mhedbi-Hajri N, Jacques MA, Koebnik R (2011) Adhesion mechanisms of plant-pathogenic *Xanthomonadaceae* . Adv Exp Med Biol 715: 71–89. 21557058 10.1007/978-94-007-0940-9_5

[95] Klosterman SJ, Subbarao KV, Kang S, Veronese P, Gold SE, et al (2011) Comparative genomics yields insights into niche adaptation of plant vascular wilt pathogens. PLoS Pathog 7: e1002137. 21829347 10.1371/journal.ppat.1002137 PMC3145793

[96] Nagy T, Emami K, Fontes CM, Ferreira LM, Humphry DR, et al (2002) The membrane-bound alpha-glucuronidase from *Pseudomonas cellulosa* hydrolyzes 4-O-methyl-D-glucuronoxylooligosaccharides but not 4-O-methyl-D-glucuronoxylan. J Bacteriol 184: 4925–4929. 12169619 10.1128/JB.184.17.4925-4929.2002 PMC135289

[97] Biely P, Vrsanska M, Tenkanen M, Kluepfel D (1997) Endo-beta-1,4-xylanase families: differences in catalytic properties. J Biotechnol 57: 151–166. 9335171 10.1016/s0168-1656(97)00096-5

[98] Sola C, Ferdinand S, Mammina C, Nastasi A, Rastogi N (2001) Genetic diversity of *Mycobacterium tuberculosis* in Sicily based on spoligotyping and variable number of tandem DNA repeats and comparison with a spoligotyping database for population-based analysis. J Clin Microbiol 39: 1559–1565. 11283087 10.1128/JCM.39.4.1559-1565.2001 PMC87970

[99] Ochiai H, Inoue Y, Takeya M, Sasaki A, Kaku H (2005) Genome sequence of *Xanthomonas oryzae* pv. *oryzae* suggests contribution of large numbers of effector genes and insertion sequences to its race diversity. Jpn Agric Res Q 39.

[100] da Silva FG, Shen Y, Dardick C, Burdman S, Yadav RC, et al (2004) Bacterial genes involved in type I secretion and sulfation are required to elicit the rice Xa21-mediated innate immune response. Mol Plant Microbe Interact 17: 593–601. 15195942 10.1094/MPMI.2004.17.6.593

[101] Szczesny R, Jordan M, Schramm C, Schulz S, Cogez V, et al (2010) Functional characterization of the Xcs and Xps type II secretion systems from the plant pathogenic bacterium *Xanthomonas campestris* pv *vesicatoria* . New Phytol 187: 983–1002. 20524995 10.1111/j.1469-8137.2010.03312.x

[102] Han M, Kim Y, Kim Y, Chung B, Choi G-W (2011) Bioethanol production from optimized pretreatment of cassava stem. Korean J Chem Eng 28: 119–125.

[103] He YW, Wu J, Zhou L, Yang F, He YQ, et al (2011) *Xanthomonas campestris* diffusible factor is 3-hydroxybenzoic acid and is associated with xanthomonadin biosynthesis, cell viability, antioxidant activity, and systemic invasion. Mol Plant Microbe Interact 24: 948–957. 21539432 10.1094/MPMI-02-11-0031

[104] Patil PB, Sonti RV (2004) Variation suggestive of horizontal gene transfer at a lipopolysaccharide (lps) biosynthetic locus in *Xanthomonas oryzae* pv. *oryzae*, the bacterial leaf blight pathogen of rice. BMC Microbiol 4: 40. 15473911 10.1186/1471-2180-4-40 PMC524487

[105] Rademaker JL, Louws FJ, Schultz MH, Rossbach U, Vauterin L, et al (2005) A comprehensive species to strain taxonomic framework for *Xanthomonas* . Phytopathology 95: 1098–1111. 18943308 10.1094/PHYTO-95-1098

[106] Myers EW, Sutton GG, Delcher AL, Dew IM, Fasulo DP, et al (2000) A whole-genome assembly of *Drosophila* . Science 287: 2196. 10731133 10.1126/science.287.5461.2196

[107] Konstantinidis KT, Tiedje JM (2005) Genomic insights that advance the species definition for prokaryotes. Proc Natl Acad Sci U S A 102: 2567–2572. 15701695 10.1073/pnas.0409727102 PMC549018

[108] Krzywinski M, Schein J, Birol I, Connors J, Gascoyne R, et al (2009) Circos: an information aesthetic for comparative genomics. Genome Res 19: 1639–1645. 19541911 10.1101/gr.092759.109 PMC2752132

[109] Lowe TM, Eddy SR (1997) tRNAscan-SE: a program for improved detection of transfer RNA genes in genomic sequence. Nucleic Acids Res 25: 955–964. 9023104 10.1093/nar/25.5.955 PMC146525

[110] Kurtz S, Phillippy A, Delcher AL, Smoot M, Shumway M, et al (2004) Versatile and open software for comparing large genomes. Genome Biol 5: R12. 14759262 10.1186/gb-2004-5-2-r12 PMC395750

[111] Schiex T, Gouzy J, Moisan A, de Oliveira Y (2003) FrameD: A flexible program for quality check and gene prediction in prokaryotic genomes and noisy matured eukaryotic sequences. Nucleic Acids Res 31: 3738–3741. 12824407 10.1093/nar/gkg610 PMC169016

[112] Delcher AL, Bratke KA, Powers EC, Salzberg SL (2007) Identifying bacterial genes and endosymbiont DNA with Glimmer. Bioinformatics 23: 673–679. 17237039 10.1093/bioinformatics/btm009 PMC2387122

[113] Li L, Stoeckert CJ Jr, Roos DS (2003) OrthoMCL: identification of ortholog groups for eukaryotic genomes. Genome Res 13: 2178–2189. 12952885 10.1101/gr.1224503 PMC403725

[114] Vernikos GS, Parkhill J (2006) Interpolated variable order motifs for identification of horizontally acquired DNA: revisiting the *Salmonella* pathogenicity islands. Bioinformatics 22: 2196. 16837528 10.1093/bioinformatics/btl369

[115] Leplae R, Hebrant A, Wodak SJ, Toussaint A (2004) ACLAME: a CLAssification of Mobile genetic Elements. Nucleic Acids Res 32: D45–49. 14681355 10.1093/nar/gkh084 PMC308818

[116] Ronquist F, Huelsenbeck JP (2003) MrBayes 3: Bayesian phylogenetic inference under mixed models. Bioinformatics 19: 1572–1574. 12912839 10.1093/bioinformatics/btg180

[117] Stamatakis A (2006) RAxML-VI-HPC: maximum likelihood-based phylogenetic analyses with thousands of taxa and mixed models. Bioinformatics 22: 2688. 16928733 10.1093/bioinformatics/btl446

[118] Edgar RC (2004) MUSCLE: multiple sequence alignment with high accuracy and high throughput. Nucleic Acids Res 32: 1792–1797. 15034147 10.1093/nar/gkh340 PMC390337

[119] Abascal F, Zardoya R, Posada D (2005) ProtTest: selection of best-fit models of protein evolution. Bioinformatics 21: 2104–2105. 15647292 10.1093/bioinformatics/bti263

[120] Jalan N, Aritua V, Kumar D, Yu F, Jones JB, et al (2011) Comparative genomic analysis of *Xanthomonas axonopodis* pv. *citrumelo* F1, which causes citrus bacterial spot disease, and related strains provides insights into virulence and host specificity. J Bacteriol 193: 6342–6357. 21908674 10.1128/JB.05777-11 PMC3209208

[121] Benson G (1999) Tandem repeats finder: a program to analyze DNA sequences. Nucleic Acids Res 27: 573–580. 9862982 10.1093/nar/27.2.573 PMC148217

[122] Qu W, Shen Z, Zhao D, Yang Y, Zhang C (2009) MFEprimer: multiple factor evaluation of the specificity of PCR primers. Bioinformatics 25: 276–278. 19038987 10.1093/bioinformatics/btn614

[123] Hunter PR, Gaston MA (1988) Numerical index of the discriminatory ability of typing systems: an application of Simpson's index of diversity. J Clin Microbiol 26: 2465–2466. 3069867 10.1128/jcm.26.11.2465-2466.1988 PMC266921

