# Supplementary figures and images for: Genomic Survey of Pathogenicity Determinants and VNTR Markers in the Cassava Bacterial Pathogen Xanthomonas axonopodis pv. Manihotis Strain CIO151
Source: PLoS One. 2013 Nov 22;8(11):e79704. doi: 10.1371/journal.pone.0079704 (PMC3838355; doi:10.1371/journal.pone.0079704)

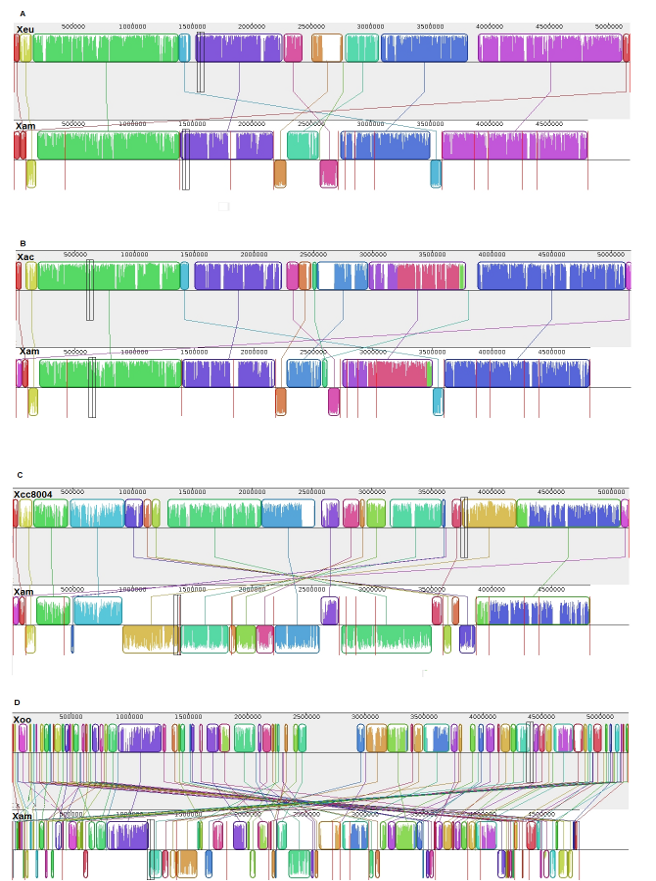

Supplement: Figure S1 — Alignment of putative chromosomal scaffolds of Xam CIO151 and Xeu, Xac, Xcc8004 and XooPXO99A chromosomes using MAUVE software. A. Alignment between Xam CIO151 and Xeu, B. Alignment between Xam and Xac, C. Alignment between Xcc8004 and Xam CIO151, D. Alignment between XooPXO99A and Xam CIO151. Vertical red lines in Xam CIO151 indicate the scaffolds. (TIFF) [file pone.0079704.s001.tiff]

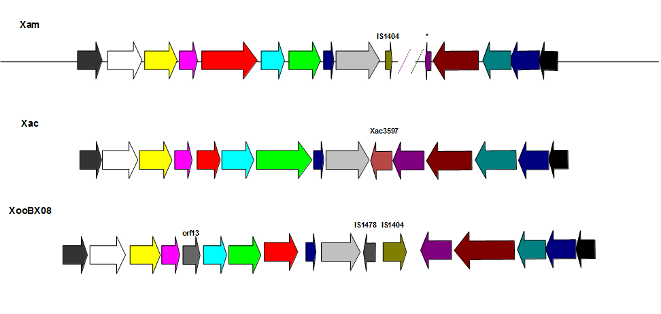

Supplement: Figure S2 — Comparison of lipopolysaccharide gene clusters of Xam, XooBX08 and Xac. Homologous genes are represented by the same color. Dotted lines in Xam indicates the distribution of the cluster on two consecutives scaffolds. (TIFF) [file pone.0079704.s002.tiff]
