# Supplementary material for: Genomic Survey of Pathogenicity Determinants and VNTR Markers in the Cassava Bacterial Pathogen Xanthomonas axonopodis pv. Manihotis Strain CIO151
Source: PLoS One. 2013 Nov 22;8(11):e79704. doi: 10.1371/journal.pone.0079704 (PMC3838355; doi:10.1371/journal.pone.0079704)
Supplement: Table S1 — Predicted proteins unique to Xam. (DOC) [file pone.0079704.s003.doc]

**Table S1. Predicted proteins unique to *Xam*.**

| **CDS name** | **Functional annotation** |
| --- | --- |
| xanmn_chr02_0026 | Hypothetical protein^$^ |
| xanmn_chr03_0199 | Hypothetical protein^$^ |
| xanmn_chr03_0203 | Hypothetical protein |
| xanmn_chr03_0204 | Hypothetical protein |
| xanmn_chr03_0209 | Hypothetical protein^$^ |
| xanmn_chr03_0212 | Hypothetical protein^$^ |
| xanmn_chr03_0217 | Hypothetical protein |
| xanmn_chr03_0245 | Hypothetical protein |
| xanmn_chr03_0299 | Hypothetical protein^$^ |
| xanmn_chr03_0300 | Hypothetical protein^$^ |
| xanmn_chr03_0305 | Hypothetical protein |
| xanmn_chr03_0309 | Conserved putative secreted protein#^$^ |
| xanmn_chr03_0311 | Hypothetical protein^$^ |
| xanmn_chr03_0322 | Hypothetical protein^$^ |
| xanmn_chr03_0347 | Hypothetical protein^$^ |
| xanmn_chr03_0456 | Hypothetical protein^$^ |
| xanmn_chr03_0505 | Hypothetical protein^$^ |
| xanmn_chr03_0508 | Hypothetical protein |
| xanmn_chr03_0528 | Hypothetical protein^$^ |
| xanmn_chr03_0532 | Hypothetical protein^$^ |
| xanmn_chr03_0603 | Hypothetical protein |
| xanmn_chr03_0729 | Hypothetical protein |
| xanmn_chr03_0745 | Hypothetical protein |
| xanmn_chr03_0765 | Hypothetical protein^$^ |
| xanmn_chr03_0833 | Hypothetical protein |
| xanmn_chr03_0836 | Putative acyltransferase |
| xanmn_chr03_0860 | Hypothetical protein^$^ |
| xanmn_chr03_0872 | Hypothetical protein^$^ |
| xanmn_chr04_0014 | Hypothetical protein^$^ |
| xanmn_chr04_0024 | Putative secreted protein#^$^ |
| xanmn_chr04_0219 | Putative membrane protein* |
| xanmn_chr04_0226 | Putative membrane protein* |
| xanmn_chr04_0227 | Putative secreted protein# |
| xanmn_chr04_0296 | Hypothetical protein |
| xanmn_chr04_0335 | Hypothetical protein |
| xanmn_chr04_0338 | Hypothetical protein^$^ |
| xanmn_chr04_0384 | Hypothetical protein^$^ |
| xanmn_chr04_0401 | Hypothetical protein |
| xanmn_chr05_0001 | Hypothetical protein |
| xanmn_chr05_0003 | Hypothetical protein |
| xanmn_chr05_0129 | Hypothetical protein^$^ |
| xanmn_chr05_0146 | Hypothetical protein^$^ |
| xanmn_chr05_0157 | Hypothetical protein^$^ |
| xanmn_chr05_0161 | Hypothetical protein^$^ |
| xanmn_chr05_0182 | Hypothetical protein^$^ |
| xanmn_chr06_0090 | Hypothetical protein^$^ |
| xanmn_chr06_0182 | Hypothetical protein^$^ |
| xanmn_chr06_0301 | Putative membrane protein* |
| xanmn_chr06_0376 | Hypothetical protein^$^ |
| xanmn_chr07_0010 | Hypothetical lipoprotein peptidase |
| xanmn_chr07_0026 | Hypothetical protein^$^ |
| xanmn_chr08_0003 | Hypothetical protein |
| xanmn_chr08_0005 | Hypothetical protein |
| xanmn_chr08_0011 | Hypothetical protein |
| xanmn_chr08_0014 | Hypothetical protein |
| xanmn_chr08_0015 | Putative membrane protein* |
| xanmn_chr09_0011 | Hypothetical protein |
| xanmn_chr09_0139 | Hypothetical protein |
| xanmn_chr10_0216 | Hypothetical protein |
| xanmn_chr10_0267 | Hypothetical histone-like protein |
| xanmn_chr10_0372 | Hypothetical protein^$^ |
| xanmn_chr10_0433 | Hypothetical protein |
| xanmn_chr10_0454 | Hypothetical protein^$^ |
| xanmn_chr10_0504 | Hypothetical protein |
| xanmn_chr10_0505 | Hypothetical protein |
| xanmn_chr10_0513 | Hypothetical protein |
| xanmn_chr10_0519 | Hypothetical protein |
| xanmn_chr11_0021 | Hypothetical protein^$^ |
| xanmn_chr11_0167 | Hypothetical protein^$^ |
| xanmn_chr11_0170 | Hypothetical protein^$^ |
| xanmn_chr11_0200 | Hypothetical protein^$^ |
| xanmn_chr11_0228 | Hypothetical protein^$^ |
| xanmn_chr11_0237 | Hypothetical protein |
| xanmn_chr12_0039 | Hypothetical protein |
| xanmn_chr12_0051 | Putative secreted protein#^$^ |
| xanmn_chr12_0063 | Hypothetical protein |
| xanmn_chr12_0071 | Hypothetical protein^$^ |
| xanmn_chr13_0003 | Hypothetical protein^$^ |
| xanmn_chr13_0031 | Hypothetical protein^$^ |
| xanmn_chr13_0037 | Hypothetical protein^$^ |
| xanmn_chr13_0050 | Putative secreted protein#^$^ |
| xanmn_chr13_0117 | Hypothetical protein^$^ |
| xanmn_chr13_0119 | Hypothetical protein^$^ |
| xanmn_chr13_0131 | Hypothetical secreted protein#^$^ |
| xanmn_chr13_5004 | D-amino acid oxidase |
| xanmn_chr13_5003 | Conserved hypothetical secreted protein# |
| xanmn_chr14_0003 | Hypothetical protein^$^ |
| xanmn_chr14_0017 | Hypothetical protein^$^ |
| xanmn_chr14_0066 | Hypothetical protein^$^ |
| xanmn_chr14_0087 | Hypothetical protein^$^ |
| xanmn_chr14_0088 | Putative blue (type 1) copper protein^$^ |
| xanmn_chr14_0125 | Hypothetical protein |
| xanmn_chr15_0089 | Hypothetical protein^$^ |
| xanmn_chr15_0094 | Hypothetical protein |
| xanmn_chr15_0095 | Hypothetical protein^$^ |
| xanmn_chr15_0138 | Hypothetical protein^$^ |
| xanmn_chr15_0173 | Conserved hypothetical protein |
| xanmn_chr15_0219 | Hypothetical protein |
| xanmn_chr15_5004 | Hypothetical protein |
| xanmn_chr15_0261 | Radical SAM domain-containing protein^$^ |
| xanmn_chr15_0263 | Conserved hypothetical protein^$^ |
| xanmn_chr15_0264 | Conserved hypothetical wgr domain-containing protein^$^ |
| xanmn_chr15_0265 | ATPase associated with various cellular activities |
| xanmn_chr15_0267 | Conserved hypothetical protein |
| xanmn_chr15_0274 | Hypothetical protein |
| xanmn_chr15_0278 | Hypothetical protein |
| xanmn_chr15_0280 | Hypothetical protein |
| xanmn_chr15_5006 | Hypothetical protein^$^ |
| xanmn_chr16_0002 | Hypothetical protein |
| xanmn_pla01_0006 | Damage inducible-like protein |
| xanmn_pla01_0007 | Putative addiction module toxin |
| xanmn_pla01_0017 | Conserved hypothetical protein |
| xanmn_pla01_0019 | Conserved hypothetical protein |
| xanmn_pla04_0007 | Hypothetical protein |
| xanmn_pla05_0004 | hypothetical protein |
| xanmn_unk01_0013 | Hypothetical protein |
| xanmn_unk01_0014 | Hypothetical protein |
| xanmn_unk08_0004 | Putative signal protein with a GGDEF domain  (N-terminal fragment) |
| xanmn_unk10_0004 | Conserved hypothetical protein |
| xanmn_unk10_0009 | Hypothetical protein |
| xanmn_unk10_0011 | Hypothetical protein |
| xanmn_unk10_0012 | Conserved hypothetical protein |
| xanmn_unk10_0020 | Hypothetical prophage antirepressor |
| xanmn_unk10_0026 | Hypothetical protein |
| xanmn_unk10_0028 | Hypothetical protein |
| xanmn_unk11_0016 | Choline dehydrogenase |

^#^ Predicted secreted protein

* Predicted membrane protein

^$^ Identified using tblastn in all 65 strains reported by Bart and collaborators [15]
