# Supplementary material for: Genomic Survey of Pathogenicity Determinants and VNTR Markers in the Cassava Bacterial Pathogen Xanthomonas axonopodis pv. Manihotis Strain CIO151
Source: PLoS One. 2013 Nov 22;8(11):e79704. doi: 10.1371/journal.pone.0079704 (PMC3838355; doi:10.1371/journal.pone.0079704)
Supplement: Table S2 — Characteristics of chromosomal regions predicted with atypical nucleotide composition. (DOC) [file pone.0079704.s004.doc]

**Table S2. Characteristics of chromosomal regions predicted with atypical nucleotide composition**.

| **Start scaffold** | **Start position** | **End scaffold** | **End position** | **Length (bp)** |
| --- | --- | --- | --- | --- |
| chr_1 | 105000 | chr_2 | 5741 | 7501 |
| chr_1 | 57500 | chr_1 | 62500 | 5001 |
| chr_1 | 87500 | chr_1 | 95000 | 7501 |
| chr_2 | 315750 | chr_3 | 1318 | 7501 |
| chr_3 | 53818 | chr_3 | 58818 | 5001 |
| chr_3 | 698818 | chr_3 | 733818 | 35001 |
| chr_3 | 796318 | chr_3 | 803818 | 7501 |
| chr_3 | 823818 | chr_3 | 828818 | 5001 |
| chr_3 | 831318 | chr_3 | 838818 | 7501 |
| chr_3 | 966318 | chr_4 | 4000 | 5001 |
| chr_3 | 76318 | chr_3 | 83818 | 7501 |
| chr_3 | 273818 | chr_3 | 281318 | 7501 |
| chr_4 | 231500 | chr_4 | 241500 | 10001 |
| chr_4 | 401500 | chr_4 | 429000 | 27501 |
| chr_5 | 1259 | chr_5 | 43759 | 42501 |
| chr_5 | 168759 | chr_5 | 176259 | 7501 |
| chr_5 | 208759 | chr_5 | 226259 | 17501 |
| chr_5 | 323759 | chr_5 | 328759 | 5001 |
| chr_5 | 353759 | chr_6 | 3991 | 7501 |
| chr_6 | 61491 | chr_6 | 66491 | 5001 |
| chr_6 | 328991 | chr_6 | 333991 | 5001 |
| chr_6 | 351491 | chr_6 | 361491 | 10001 |
| chr_6 | 386491 | chr_6 | 393991 | 7501 |
| chr_6 | 406491 | chr_6 | 418991 | 12501 |
| chr_6 | 513991 | chr_6 | 528991 | 15001 |
| chr_7 | 4277 | chr_7 | 11777 | 7501 |
| chr_8 | 5545 | chr_8 | 10545 | 5001 |
| chr_8 | 33045 | chr_8 | 38045 | 5001 |
| chr_8 | 63045 | chr_8 | 83045 | 20001 |
| chr_8 | 85545 | chr_9 | 11850 | 12501 |
| chr_9 | 29350 | chr_9 | 36850 | 7501 |
| chr_9 | 84350 | chr_9 | 91850 | 7501 |
| chr_10 | 263082 | chr_10 | 268082 | 5001 |
| chr_10 | 555582 | chr_10 | 565582 | 10001 |
| chr_11 | 64243 | chr_11 | 69243 | 5001 |
| chr_11 | 136743 | chr_11 | 144243 | 7501 |
| chr_12 | 18220 | chr_12 | 23220 | 5001 |
| chr_12 | 38220 | chr_12 | 43220 | 5001 |
| chr_12 | 105720 | chr_13 | 7130 | 12501 |
| chr_13 | 284630 | chr_14 | 7108 | 10001 |
| chr_14 | 44609 | chr_14 | 59609 | 15001 |
| chr_14 | 117108 | chr_15 | 1613 | 7501 |
| chr_15 | 19114 | chr_15 | 26614 | 7501 |
| chr_15 | 64114 | chr_15 | 71614 | 7501 |
| chr_15 | 119114 | chr_15 | 124114 | 5001 |
| chr_15 | 161614 | chr_15 | 166614 | 5001 |
| chr_15 | 269114 | chr_15 | 274114 | 5001 |
| chr_15 | 329114 | chr_15 | 336614 | 7501 |
| chr_15 | 371614 | chr_15 | 376614 | 5001 |
| chr_15 | 379115 | chr_15 | 384115 | 5001 |
