# Supplementary material for: Genomic Survey of Pathogenicity Determinants and VNTR Markers in the Cassava Bacterial Pathogen Xanthomonas axonopodis pv. Manihotis Strain CIO151
Source: PLoS One. 2013 Nov 22;8(11):e79704. doi: 10.1371/journal.pone.0079704 (PMC3838355; doi:10.1371/journal.pone.0079704)
Supplement: Table S3 — Potential pathogenicity associated islands (PAIs) in Xam. (DOC) [file pone.0079704.s005.doc]

**Table S3. Potential pathogenicity associated islands (PAIs) in *Xam***

^&^ automatically annotated pseudogenes
