# Supplementary material for: Genomic Survey of Pathogenicity Determinants and VNTR Markers in the Cassava Bacterial Pathogen Xanthomonas axonopodis pv. Manihotis Strain CIO151
Source: PLoS One. 2013 Nov 22;8(11):e79704. doi: 10.1371/journal.pone.0079704 (PMC3838355; doi:10.1371/journal.pone.0079704)
Supplement: Table S4 — rpf gene cluster of Xam CIO151. (DOCX) [file pone.0079704.s006.docx]

**Table S4. *rpf* gene cluster of *Xam* CIO151.**

| **Gene name** | **CDS name** |
| --- | --- |
| *rpfE* | xanmn_chr05_0232 |
| *recJ* | xanmn_chr05_0233 |
| PMP | xanmn_chr05_0234 |
| *rpfD* | xanmn_chr05_0235 |
| *lysS* | xanmn_chr05_0236 |
| *rpfG* | xanmn_chr05_0237 |
| *rpfH* | xanmn_chr05_0238 |
| *rpfC* | xanmn_chr05_0239 |
| *rpfF* | xanmn_chr05_0240 |
| *rpfB* | xanmn_chr05_0241 |
| CHP^b^ | xanmn_chr05_0242 |
| *rpfA* | xanmn_chr05_0243 |

^a^ PMP, putative membrane protein.

^b^ CHP, conserved hypothetical protein.
