# Supplementary material for: Genomic Survey of Pathogenicity Determinants and VNTR Markers in the Cassava Bacterial Pathogen Xanthomonas axonopodis pv. Manihotis Strain CIO151
Source: PLoS One. 2013 Nov 22;8(11):e79704. doi: 10.1371/journal.pone.0079704 (PMC3838355; doi:10.1371/journal.pone.0079704)
Supplement: Table S5 — Type II secretion systems in Xam CIO151. (DOC) [file pone.0079704.s007.doc]

**Table S5. Type II secretion systems in *Xam* CIO151.**

| **Gene name** | **CDS name** |
| --- | --- |
| *xpsD* | xanmn_chr12_0088 |
|  | xanmn_chr12_0089# |
| *xpsM* | xanmn_chr12_0019 |
| *xpsN* | £ |
| *xpsL* | xanmn_chr12_0020# |
|  | xanmn_chr12_0021# |
| *xpsK* | xanmn_chr12_0022 |
| *xpsJ* | xanmn_chr12_0023 |
| *xpsI* | xanmn_chr12_0024 |
| *xpsH* | xanmn_chr12_0090* |
|  | xanmn_chr12_0091* |
| *xpsG* | xanmn_chr12_0025 |
| *xpsF* | xanmn_chr12_0026 |
| *xpsE* | xanmn_chr12_0027 |
| *xcsC* | xanmn_chr03_0407 |
| *xcsD* | xanmn_chr03_0408 |
| *xcsE* | xanmn_chr03_0409 |
| *xcsF* | xanmn_chr03_0410 |
| *xcsG* | xanmn_chr03_0411 |
| *xcsH* | xanmn_chr03_0412 |
| *xcsI* | xanmn_chr03_0413 |
| *xcsJ* | xanmn_chr03_0414 |
| *xcsK* | xanmn_chr03_0415 |
| *xcsL* | xanmn_chr03_0416 |
| *xcsM* | xanmn_chr03_0417 |
| *xcsN* | xanmn_chr03_0418 |

^#^ Fragmented by indeterminate nucleotide positions.

* Potential pseudogenes.

£ Not predicted due to indeterminate nucleotide positions.
