# Supplementary material for: Genomic Survey of Pathogenicity Determinants and VNTR Markers in the Cassava Bacterial Pathogen Xanthomonas axonopodis pv. Manihotis Strain CIO151
Source: PLoS One. 2013 Nov 22;8(11):e79704. doi: 10.1371/journal.pone.0079704 (PMC3838355; doi:10.1371/journal.pone.0079704)
Supplement: Table S6 — Type III secretion system (hrp cluster) in Xam CIO151. (DOCX) [file pone.0079704.s008.docx]

**Table S6. Type III secretion system (*hrp* cluster) in *Xam* CIO151.**

| **Gene name** | **CDS name** |
| --- | --- |
| *hpaF* | xanmn_chr03_0052 |
| *hrpF* | xanmn_chr03_0053 |
| HP | xanmn_chr03_0054 |
| *hpa3* | xanmn_chr03_0056 |
| *xopF1^#^* | xanmn_chr03_0057 |
| HP | xanmn_chr03_0058 |
| *hpaB* | xanmn_chr03_5025 |
| *hrpE* | xanmn_chr03_0060 |
| *hrpD6* | xanmn_chr03_0061 |
| *hrcD* | xanmn_chr03_0062 |
| *hpaA* | xanmn_chr03_5026 |
| *hrcS* | xanmn_chr03_0063 |
| *hrcR* | xanmn_chr03_0064 |
| *hrcQ* | xanmn_chr03_0065 |
| *hpaC* | xanmn_chr03_5027 |
| *hrcV* | xanmn_chr03_0066 |
| *hrcU* | xanmn_chr03_0067 |
| *hrpB1* | xanmn_chr03_0068 |
| *hrpB2* | xanmn_chr03_0069 |
| *hrcJ* | xanmn_chr03_0070 |
| *hrpB4* | xanmn_chr03_5028 |
| *hrcL* | xanmn_chr03_0071 |
| *hrcN* | xanmn_chr03_0072 |
| *hrpB7* | xanmn_chr03_0073 |
| *hrcT* | xanmn_chr03_0074 |
| *hrcC* | xanmn_chr03_0075 |
| *hpa1* | xanmn_chr03_0076 |
| *hpa2* | xanmn_chr03_0077 |

^#^ Potential pseudogene.

HP = hypothetical protein.
