# Supplementary material for: Genomic Survey of Pathogenicity Determinants and VNTR Markers in the Cassava Bacterial Pathogen Xanthomonas axonopodis pv. Manihotis Strain CIO151
Source: PLoS One. 2013 Nov 22;8(11):e79704. doi: 10.1371/journal.pone.0079704 (PMC3838355; doi:10.1371/journal.pone.0079704)
Supplement: Table S7 — Xanthan gum gene cluster in Xam CIO151. (DOC) [file pone.0079704.s009.doc]

**Table S7. Xanthan *gum* gene cluster in *Xam* CIO151.**

| **Gene name** | **CDS name** |
| --- | --- |
| *cdh2* | xanmn_chr08_0024 |
| *gumP* | xanmn_chr08_0025 |
| *fabH(gumO)* | xanmn_chr08_0026 |
| *gumN* | xanmn_chr08_0027 |
| HP | xanmn_chr08_0028 |
| *gumM* | xanmn_chr08_0029 |
| *gumL* | xanmn_chr08_0030 |
| *gumK* | xanmn_chr08_0031 |
| *gumJ* | xanmn_chr08_0032 |
| *gumI* | xanmn_chr08_0033 |
| *gumH* | xanmn_chr08_0034 |
| *gumG* | xanmn_chr08_0035 |
| *gumF* | xanmn_chr08_0036 |
| *gumE* | xanmn_chr08_0037 |
| *gumD* | xanmn_chr08_0038 |
| *gumC* | xanmn_chr08_0039 |
| *gumB* | xanmn_chr08_0040 |
| xanmn_chr08_0041 | xanmn_chr08_0041 |
| *gumA* | xanmn_chr08_0042 |

HP = hypothetical protein.
