# Supplementary material for: Genomic Survey of Pathogenicity Determinants and VNTR Markers in the Cassava Bacterial Pathogen Xanthomonas axonopodis pv. Manihotis Strain CIO151
Source: PLoS One. 2013 Nov 22;8(11):e79704. doi: 10.1371/journal.pone.0079704 (PMC3838355; doi:10.1371/journal.pone.0079704)
Supplement: Table S8 — CDS of putative effector proteins detected in the genome of Xam CIO151. (DOCX) [file pone.0079704.s010.docx]

**Table S8. CDS of putative effector proteins detected in the genome of *Xam* CIO151.**

| **Effector family** | **CDS name** |
| --- | --- |
| AvrBs2 | xanmn_chr02_0062 |
| TAL | 2* |
| XopC2 | xanmn_chr10_0520 |
| XopE1 (HopX) | xanmn_chr02_0208 |
| XopE4 (HopX) | xanmn_pla04_0019 |
| XopF | xanmn_chr03_0057# |
| XopK | xanmn_chr10_0294 |
| XopL | xanmn_chr10_0299 |
| XopN | xanmn_chr09_5006 |
| XopP | xanmn_chr10_0524# |
|  | xanmn_chr10_0523# |
|  | xanmn_chr10_0522# |
|  | xanmn_chr10_0521# |
| XopQ (HopQ1) | xanmn_chr15_5505 |
| XopR | xanmn_chr02_0199 |
| XopV | xanmn_chr03_0302 |
| XopX | xanmn_chr03_0215 |
| XopZ (HopAS1) | xanmn_chr06_0393 |
| XopAD (SKWP) | xanmn_chr15_0210# |
|  | xanmn_chr15_0209# |
|  | xanmn_chr15_0208# |
| XopAE (HpaF) | xanmn_chr03_0052 |
| XopAG (HopG1) | xanmn_chr02_0124# |
|  | xanmn_chr02_0123# |
| XopAK (HopK1) | xanmn_chr13_0074 |
| XopAO | xanmn_chr06_5019 |
|  | xanmn_chr07_0047 |

^#^ Possible pseudogene.

* Inferred from N- and C-terminal ends, not assembled.
