# Supplementary material for: Genomic Survey of Pathogenicity Determinants and VNTR Markers in the Cassava Bacterial Pathogen Xanthomonas axonopodis pv. Manihotis Strain CIO151
Source: PLoS One. 2013 Nov 22;8(11):e79704. doi: 10.1371/journal.pone.0079704 (PMC3838355; doi:10.1371/journal.pone.0079704)
Supplement: Table S9 — Putative set of cell wall-degrading enzymes present in Xam CIO151. (DOCX) [file pone.0079704.s011.docx]

**Table S9. Putative set of cell wall-degrading enzymes present in *Xam* CIO151.**

| **Protein family** | **CDS name** |
| --- | --- |
| Cellulase | xanmn_chr01­_0077* |
| Cellulase | xanmn_chr01_0079* |
| Cellulase | xanmn_chr01_0081* |
| Cellulase | xanmn_chr03_0001 |
| Cellulase | xanmn_chr03_0296 |
| Cellulase | xanmn_chr05_0133 |
| Cellulase | xanmn_chr06_0316 |
| Cellulase | xanmn_chr11_0232^a^ |
| Cellulase | xanmn_chr11_0233^a^ |
| Xylanase | xanmn_chr03_0633 |
| Xylanase | xanmn_chr07_0024 |
| Xylanase | xanmn_chr15_0050 |
| Xylanase | xanmn_chr15_0238 |
| Xylanase | xanmn_chr15_0240 |
| Xylanase | xanmn_chr15_0354^#^ |
| Xylanase | xanmn_chr15_0355^#^ |
| Pectate lyase | xanmn_chr10_0199^b^ |
| Pectate lyase | xanmn_chr10_0200^b^ |
| Pectate lyase | xanmn_chr12_0050 |
| Endoglucanase | xanmn_chr03_0318 |
| Endoglucanase | xanmn_chr12_0002 |
| beta-glucosidase | xanmn_chr04_0171 |
| beta-glucosidase | xanmn_chr05_0158 |
| beta-glucosidase | xanmn_chr10_0289 |
| beta-glucosidase | xanmn_chr13_0268 |
| beta-glucosidase | xanmn_chr15_0226 |
| alpha-glucosidase | xanmn_chr05_0136 |
| alpha-glucosidase | xanmn_chr06_0472^#^ |
| alpha-glucosidase | xanmn_chr08_0052 |
| alpha-glucosidase | xanmn_chr08_0053 |
| alpha-glucosidase | xanmn_chr08_0056 |
| alpha-glucosidase | xanmn_chr11_0024 |
| Polygalacturonase | xanmn_chr03_0374 |
| Rhamnogalacturonase | xanmn_chr02_0086 |
| Rhamnogalacturonase | xanmn_chr11_0230 |

^#^ Possible pseudogene.

* Located in region with atypical nucleotide composition.

^a,b^ Splitted sequences due to indeterminate sequence stretches are indicated by the same character.
