# Supplementary material for: Genomic Survey of Pathogenicity Determinants and VNTR Markers in the Cassava Bacterial Pathogen Xanthomonas axonopodis pv. Manihotis Strain CIO151
Source: PLoS One. 2013 Nov 22;8(11):e79704. doi: 10.1371/journal.pone.0079704 (PMC3838355; doi:10.1371/journal.pone.0079704)
Supplement: Table S10 — Oligonucleotide primers, PCR conditions, and characteristics of VNTRs analyzed in this study. (DOCX) [file pone.0079704.s012.docx]

**Table S10. Oligonucleotide primers, PCR conditions, and characteristics of VNTRs analyzed in this study.**

|  | **PCR conditions^1^** | | | **Expected amplicon size (bp) for strain^2^** | | | |
| --- | --- | --- | --- | --- | --- | --- | --- |
| **VNTR locus** | **Primers (5’-3’)** | **Temp (°C)** | **Time (s)** | ***Xam* CIO151** | ***Xac***  **306** | ***Xav***  **85-10** | ***Xac***  **F1** |
| XaG1_02 | GCTGGTGCGCATGCTGAC  TTCGCGGAAGGTCATGGG | 55 | 60 | 362 | 430 | 356 | 391 |
| XaG1_12 | GCATCAGGCCGCACGCTT  GATGTGCGCGATGCGGTG | 52 | 30 | 111 | NA^3^ | 111 | 111 |
| XaG1_29 | CTACGAGGCACCTGCGCA  CATGGCATCGCGCTGAAG | 52 | 60 | 251 | 207 | 201 | 201 |
| XaG1_58 | GCACGCGGTTGCAGTAGC  TGACCTTGTTGCGGACGC | 52 | 45 | 192 | 156 | 162 | 168 |
| XaG1_65 | CGCCGCCACATCAGGTTG  GTTGGTGCTGCTTCGGGG | 52 | 60 | 402 | NA | 417 | 417 |
| XaG1_67 | GTCGGCGCGATCGTCATG  GTTGCGGCACGCCAACCT | 53 | 30 | 446 | 359 | 344 | NA |
| XaG1_70 | TGAAGCTGGTGGTGCGCG  AGCGGCGATGCTGTGCGT | 52 | 60 | 344 | 234 | 281 | NA |
| XaG1_71 | GGCCAGTGTACGAGTGGCG  TTGACACATTCGCCGTCGA | 52 | 30 | 184 | 156 | 151 | 152 |
| XaG1_72 | CGAAGCGATCGGCTACCG  CAGGCCTCTGCACAATGGAG | 52 | 30 | 402 | NA | 396 | 395 |
| XaG1_73 | CACGCCCAAGCCCATCCA  CACCGAGGTCGCGGCATC | 52 | 60 | 268 | 277 | 256 | 256 |
| XaG2_50 | TGATCTGCTCAGGCGTGGC  GATGCTTGGCAGCGTGTCTG | 52 | 30 | 119 | 107 | 107 | 101 |
| XaG1_101 | AGGCGTTGTATCGGTTGAAG  AATCGATTTCCGATTCCTG |  |  | NA | NA | NA | NA |
| XaG1_105 | GCGCTGCGATTGCCATAG  CGCTTGCCGAGTCGCAAC |  |  | 142 | NA | NA | NA |
| XaG1_108 | TGCGCTCGTCGGCTACCA  TATGTGCAGATCAACTACCAC |  |  | 163 | NA | NA | NA |
| XaG1_110 | TGATGACACTGCCGCAGT  AGTGTTCTCTTGATGACTCA |  |  | 150 | NA | NA | NA |
| XaG2_37 | CACCGCGCAACCTGGTCA  CTACAGCGCCGGCGACG | 58 | 30 | 548 | 528 | 532 | 532 |
| XaG2_52 | GTCGTTCAATCCGGGCGA  GGCGATTTCGGGCTGAGG | 52 | 30 | 321 | NA | 188 | 193 |
| XaG2_55 | GTCAACGCCAGCATCAACCA  GTATGGGGCCCCGAAAGC | 52 | 30 | 253 | 256 | 253 | 238 |
| XaG2_106 | CATCGCGAGCAGGTCGCT  CGAGTCGTGGCAGTTGAG |  |  | 157 | NA | NA | NA |
| XaG2_109 | CAACAGAAGATGCTCAAA  CTTCTGGGAATCGAGAGG |  |  | NA | NA | NA | NA |
| XaG2_116 | GCACCGAAATACACCAAT  TACGATCGGCTGGAGGTG |  |  | 123 | NA | NA | NA |
| XaG2_117 | GCAAGCGGGAGAAGGAAG  CGGTGGAAGCGGTGTTCG |  |  | 178 | NA | NA | NA |

^1^ PCR conditions (Temp=annealing temperature; Time=elongation time) are given.

^2^ Predicted amplicon sizes (in bp) for selected *Xanthomonas* strains.

^3^ NA, no ampicon *in silico*.
