# Supplementary material for: Genomic Survey of Pathogenicity Determinants and VNTR Markers in the Cassava Bacterial Pathogen Xanthomonas axonopodis pv. Manihotis Strain CIO151
Source: PLoS One. 2013 Nov 22;8(11):e79704. doi: 10.1371/journal.pone.0079704 (PMC3838355; doi:10.1371/journal.pone.0079704)
Supplement: Table S11 — List of Xam strains used to evaluate VNTR primers. (DOCX) [file pone.0079704.s013.docx]

**Table S11. List of *Xam* strains used to evaluate VNTR primers.**

| **Strain** | **Origin** | **Year of isolation** | **Reference** |
| --- | --- | --- | --- |
| CIAT1135 | Taiwan | 1975 | [[27](#_ENREF_27),[126](#_ENREF_126)] |
| CIAT1171 | Thailand | 1977 |  |
| CIAT1211 | New Zealand | 1980 |  |
| ORST10 | Congo | 1977 |  |
| ORST216 | Cameroon |  | [[126](#_ENREF_126)] |
| ORST241 | Uganda |  | [[126](#_ENREF_126)] |
| ORST242 | Uganda |  | [[126](#_ENREF_126)] |
| CIAT1241 | Argentina | 1984 | [[126](#_ENREF_126)] |
| NCPPB1159 | Brazil | 1941 | [[27](#_ENREF_27),[126](#_ENREF_126)] |
| CIAT1202 | Colombia | 1981 | [[27](#_ENREF_27)] |
| CIO1256 | Colombia | before 1995 |  |
| CIO25 | Colombia | before (?) 1995 | [[27](#_ENREF_27)] |
| CIO26 | Colombia | before (?) 1995 | [[27](#_ENREF_27)] |
| CIO151 | Colombia | 1995 | [[27](#_ENREF_27)] |
