# Supplementary material for: Genomic Survey of Pathogenicity Determinants and VNTR Markers in the Cassava Bacterial Pathogen Xanthomonas axonopodis pv. Manihotis Strain CIO151
Source: PLoS One. 2013 Nov 22;8(11):e79704. doi: 10.1371/journal.pone.0079704 (PMC3838355; doi:10.1371/journal.pone.0079704)
Supplement: Table S12 — Presence and characteristics of VNTR markers in 65 genome sequences of Xam worldwide. (PDF) [file pone.0079704.s014.pdf]

Table S12. Presence and characteristics of VNTR markers in 65 genome sequences of *Xam* worldwide

Strain	Origin	Year	N50	Contigs	Code
	**Data are taken from Table S1 of the publication Bart et al., 2012, Proc. Natl. Acad. Sci. U.S.A. 109(28): E1972–E1979.**				**Number of Genbank entry**
AFNC1360	Benin/	1998	25	150	AKCW01000000
AT6B	Venezuela	2006	22	163	AKCX01000000
CFBP1851	Colombia	1974	17	123	AKCY01000000
CIO1	Colombia	1995	19	128	AKCZ01000000
CIO151	Colombia	1995	24	175	AKDA01000000
IBSBF1182	Brazil	1995	128	648	AKDB01000000
IBSBF1411	Brazil	1998	15	130	AKDC01000000
IBSBF1994	Brazil	2004	137	677	AKDD01000000
IBSBF2345	Brazil	2006	19	128	AKDE01000000
IBSBF2346	Brazil	2006	18	122	AKDF01000000
IBSBF2538	Brazil	2007	122	573	AKDG01000000
IBSBF2539	Brazil	2007	24	138	AKDH01000000
IBSBF2665	Brazil	2009	83	434	AKDI01000000
IBSBF2666	Brazil	2009	49	311	AKDJ01000000
IBSBF2667	Brazil	2009	21	138	AKDK01000000
IBSBF2670	Brazil	2009	17	139	AKDL01000000
IBSBF2672	Brazil	2009	18	131	AKDM01000000
IBSBF2673	Brazil	2009	53	305	AKDN01000000
IBSBF278	Brazil	1965	20	138	AKDO01000000
IBSBF280	Brazil	1973	171	850	AKDP01000000
IBSBF2816	Brazil	2010	19	145	AKDQ01000000
IBSBF2818	Brazil	2010	22	139	AKDR01000000
IBSBF2819	Brazil	2010	104	557	AKDS01000000
IBSBF2820	Brazil	2010	19	150	AKDT01000000
IBSBF2821	Brazil	2010	21	161	AKDU01000000
IBSBF2822	Brazil	2010	93	464	AKDV01000000
IBSBF285	Nigeria	1973	21	147	AKDW01000000
IBSBF289	Brazil	?	23	155	AKDX01000000
IBSBF320	Brazil	1974	17	121	AKDY01000000
IBSBF321	Brazil	1974	183	888	AKDZ01000000
IBSBF356	Brazil	1980	18	130	AKEA01000000
IBSBF436	Brazil	1982	19	149	AKEB01000000
IBSBF614	Brazil	1987	136	706	AKEC01000000
IBSBF725	Brazil	1988	19	141	AKED01000000
IBSBF726	Brazil	1988	21	140	AKEE01000000
NCPPB1159[1]	Brazil ?[1]	1941 ?[1]	17	128	AKEF01000000
NG1	Nigeria	2011	160	785	AKEG01000000
ORST17	Congo	1984	23	177	AKEH01000000
ORST4	Colombia	1974	161	767	AKEI01000000
ORSTX27	Togo	1989	21	133	AKEJ01000000
ThaiXam	Thailand	1997	62	324	AKEK01000000
UA226	Colombia	2008	18	129	AKEL01000000

UA303	Colombia	2008	23	130	AKEM01000000
UA306	Colombia	2008	23	144	AKEN01000000
UA323	Colombia	2008	23	151	AKEO01000000
UA324	Colombia	2008	22	146	AKEP01000000
UA536	Colombia	2009	23	142	AKEQ01000000
UA556	Colombia	2009	22	139	AKER01000000
UA560	Colombia	2009	27	154	AKES01000000
UA686	Colombia	2009	22	143	AKET01000000
UG21	Uganda	2011	23	148	AKEU01000000
UG23	Uganda	2011	22	133	AKEV01000000
UG24	Uganda	2011	20	145	AKEW01000000
UG27	Uganda	2011	19	131	AKEX01000000
UG28	Uganda	2011	131	676	AKEY01000000
UG39	Uganda	2011	21	130	AKEZ01000000
UG43	Uganda	2011	23	145	AKFA01000000
UG44	Uganda	2011	24	141	AKFB01000000
UG45	Uganda	2011	59	323	AKFC01000000
UG51	Uganda	2011	22	140	AKFD01000000
Xam1134	?	/	28	158	AKFE01000000
Xam668	Indonesia	1978	29	195	AKFF01000000
Xam669	Brazil	1973	21	137	AKFG01000000
Xam672	Cameroon	1976	25	165	AKFH01000000
Xam678	DR Congo	1973	47	256	AKFI01000000

[1] Origin and year are not supported by the NCPPB strain collection entry at http://www.ncppb.com/

XaG1_02	XaG1_12	XaG1_29	XaG1_58	XaG1_65	XaG1_67
**7 bp**	**7 bp**	**7 bp**	**6 bp**	**6 bp**	**6 bp**
8	4	10	3	7	9
20	9	22	9	12	22
48	63	48	65	65	51
12	5	10	7	6	12
**0.8945**	**0.6354**	**0.8537**	**0.6582**	**0.6697**	**0.9004**
10	5	13	6	9	13
13	4	14	4	10	14
16	5	12	4	8	17
8	9	14	3	9	13
nd	6	12	4	8	17
nd	5	nd	4	12	20
12	6	nd	4	9	19
14	5	nd	4	9	22
16	5	12	4	8	nd
16	5	12	4	8	21
19	5	19	6	8	22
20	5	nd	6	8	nd
11	5	nd	4	9	nd
nd	5	nd	4	9	19
10	5	14	4	9	21
12	7	21	4	9	20
13	5	19	4	9	19
nd	5	19	4	9	nd
12	5	13	7	12	16
nd	5	nd	7	7	nd
9	6	19	4	9	19
nd	4	14	6	9	19
nd	5	nd	4	10	19
13	nd	18	4	9	19
nd	6	nd	4	11	nd
10	5	22	4	9	18
14	4	nd	5	9	21
16	5	14	4	9	nd
17	5	nd	4	10	20
15	5	nd	4	10	18
16	6	14	4	9	22
15	5	18	4	9	nd
nd	5	nd	4	9	nd
nd	5	14	4	10	14
10	5	15	4	11	16
nd	6	12	4	8	17
nd	4	12	7	8	14
12	4	13	6	9	13
17	5	12	4	8	nd
12	4	13	6	10	13
nd	nd	nd	6	8	nd
15	5	10	4	9	15
12	6	15	7	8	14
12	4	13	6	9	13
13	5	13	7	8	15

14	5	nd	8	8	15
14	6	15	7	8	15
nd	4	14	6	9	19
14	5	15	7	7	12
15	6	16	7	7	14
16	4	13	6	9	13
16	4	15	6	9	14
15	4	14	6	9	9
15	4	14	6	9	9
11	4	13	6	9	14
12	6	15	6	9	14
16	4	15	6	9	14
14	4	13	6	9	14
nd	4	15	6	9	nd
13	4	14	6	12	16
12	4	12	6	9	13
nd	5	18	9	12	nd
nd	5	nd	4	10	nd
11	5	nd	7	12	16
13	4	13	6	9	13

XaG1_70	XaG1_71	XaG1_72	XaG1_73	XaG1_101	XaG1_105
**7 bp**	**6 bp**	**6 bp**	**6 bp**	**7 bp**	**8 bp**
10	3	4	3	4	3
15	12	11	16	6	11
39	57	59	60	63	64
6	9	8	12	3	8
**0.7733**	**0.7782**	**0.7095**	**0.9034**	**0.6175**	**0.6935**
nd	7	10	14	5	10
11	nd	nd	8	5	4
10	6	5	12	5	3
10	6	5	7	4	8
10	3	5	12	5	3
12	nd	7	10	4	3
nd	6	6	9	5	3
nd	6	6	7	5	3
nd	6	5	8	4	3
10	8	5	8	5	3
nd	7	8	10	4	6
nd	7	7	13	4	5
14	8	5	11	4	3
12	7	6	nd	5	3
14	6	5	11	5	3
nd	7	6	8	6	3
nd	6	6	12	4	3
13	6	6	12	4	3
12	7	6	13	5	3
13	4	6	9	4	5
nd	5	6	8	5	3
13	nd	7	10	4	6
nd	6	6	8	5	3
nd	6	6	7	5	3
nd	7	6	8	5	3
13	7	6	10	4	3
13	7	6	11	4	3
nd	7	5	9	4	3
nd	6	7	12	4	3
nd	5	6	6	5	3
14	8	5	9	4	3
14	6	6	7	5	3
14	5	6	6	5	3
nd	10	7	nd	5	3
nd	9	7	14	4	3
10	6	5	8	5	3
12	nd	nd	16	4	9
nd	12	11	13	5	8
nd	nd	5	8	5	3
13	7	7	12	5	10
nd	6	4	10	5	5
nd	4	7	14	5	5
14	5	6	3	6	4
13	nd	4	13	5	8
14	5	6	3	4	4

14	5	7	3	4	4
15	3	6	3	6	4
13	6	7	10	4	6
nd	5	6	3	6	3
14	6	6	3	6	4
12	5	5	nd	6	6
13	6	nd	13	5	10
nd	6	6	11	5	8
nd	6	6	11	4	8
nd	nd	7	12	nd	9
13	7	7	10	5	11
13	7	7	13	6	10
14	5	nd	nd	6	9
nd	6	nd	13	nd	nd
13	7	7	15	6	9
13	10	9	10	5	9
12	7	6	12	5	3
15	10	7	12	4	3
12	7	6	nd	4	3
13	nd	nd	12	4	10

XaG1_108	XaG1_110	XaG2_37	XaG2_50	XaG2_52	XaG2_55
**6 bp**	**21 bp**	**24 bp**	**6 bp**	**13 bp**	**12 bp**
3	2	1	5	4	2
4	4	2	12	10	5
65	59	64	61	52	65
2	3	2	7	7	3
**0.3058**	**0.5231**	**0.4762**	**0.7738**	**0.8386**	**0.1462**
3	2	1	5	9	3
3	2	1	7	9	3
4	3	2	5	5	2
4	4	1	8	6	5
4	3	2	5	5	2
3	3	2	6	nd	3
3	3	1	10	8	3
3	3	1	nd	nd	3
4	3	2	9	8	3
4	3	2	9	8	3
3	nd	2	7	7	3
3	2	2	6	4	3
4	nd	2	7	nd	3
3	3	1	9	nd	3
4	3	2	7	9	3
3	3	1	8	7	3
3	3	1	7	7	3
3	3	1	7	nd	3
3	2	1	5	7	3
3	2	1	10	nd	3
3	3	1	7	6	3
3	2	1	7	10	3
3	nd	1	5	nd	3
3	3	1	9	9	3
3	3	1	nd	nd	3
3	3	1	7	nd	3
3	2	1	8	6	3
4	3	2	7	10	3
3	3	2	5	8	3
3	3	nd	7	7	3
4	3	2	8	10	3
3	3	1	8	8	3
3	3	1	7	nd	3
3	3	2	8	8	3
3	3	1	7	9	3
4	3	2	5	5	2
3	2	1	6	7	3
3	2	1	5	10	3
4	nd	2	5	5	2
3	2	1	5	6	3
3	nd	2	nd	6	3
4	3	2	7	9	3
3	2	2	6	7	3
3	2	1	5	10	3
3	2	2	12	7	3

3	2	2	5	5	3
3	2	2	8	8	3
3	2	1	7	10	3
3	2	2	5	9	3
3	2	2	5	8	3
3	2	1	6	9	3
3	2	1	6	9	3
3	2	1	5	9	3
3	2	1	5	9	3
3	nd	1	nd	10	3
3	2	1	5	9	3
3	2	1	6	9	3
3	2	1	5	nd	3
3	2	1	5	4	3
3	2	1	5	7	3
3	2	1	7	9	3
3	2	1	8	7	3
3	3	2	5	8	3
3	2	1	5	nd	3
3	2	1	6	nd	3

**XaG2_106**	**XaG2_109**	**XaG2_116**	**XaG2_117**	**Locus**
**22 bp**	**26 bp**	**22 bp**	**25 bp**	**Repeat unit size**
2	2	1	1	Minimal number of repeats
3	3	2	2	Maximal number of repeats
65	56	65	64	Number of samples
2	2	2	2	Number of haplotypes
**0.1702**	**0.4857**	**0.0894**	**0.0313**	**HGDI score**
2	2	2	2	
2	2	2	2	
2	2	2	2	
2	2	2	1	
2	2	2	2	
2	3	2	2	
2	3	2	2	
2	3	2	2	
2	3	2	2	
2	3	2	2	
2	nd	2	2	
2	2	2	2	
3	3	2	2	
2	3	2	2	
3	3	2	2	
2	3	2	2	
2	nd	2	2	
2	3	2	2	
3	2	2	2	
2	2	2	2	
2	3	2	2	
2	2	2	2	
2	3	2	2	
2	3	2	nd	
2	3	2	2	
2	3	2	2	
2	2	1	2	
3	nd	2	2	
2	3	2	2	
2	3	2	2	
3	3	2	2	
2	3	2	2	
2	3	2	2	
2	3	2	2	
2	3	2	2	
2	2	2	2	
2	2	2	2	
2	2	2	2	
2	2	2	2	
2	2	2	2	
2	2	1	2	
2	2	2	2	
2	2	2	2	
2	2	2	2	
2	nd	2	2	

2	2	2	2
2	2	2	2
2	2	2	2
2	nd	2	2
2	2	2	2
2	2	1	2
2	nd	2	2
2	2	2	2
2	2	2	2
2	nd	2	2
2	2	2	2
2	2	2	2
2	2	2	2
2	nd	2	2
2	2	2	2
2	2	2	2
2	2	2	2
2	nd	2	2
3	2	2	2
2	2	2	2
